# Supplementary material for: Integrating endogenous TurboID and data-independent acquisition mass spectrometry for in vivo proximity labeling
Source: EMBO J. 2025 Dec 11;45(2):592–632. doi: 10.1038/s44318-025-00660-5 (PMC12811337; doi:10.1038/s44318-025-00660-5)
Supplement: Supplementary file 1 — Appendix [file 44318_2025_660_MOESM1_ESM.pdf]

Appendix

Table of Contents

Figure S1...2
(western blot images)

Supplementary Methods S1...13
(sequencing information for N- and C-terminal fusions of TurboID)

Supplementary Methods S2...16
(LC-MS/MS experimental details)

Appendix Figure S1. Western blot data for Exp 1–8

Experiment 1

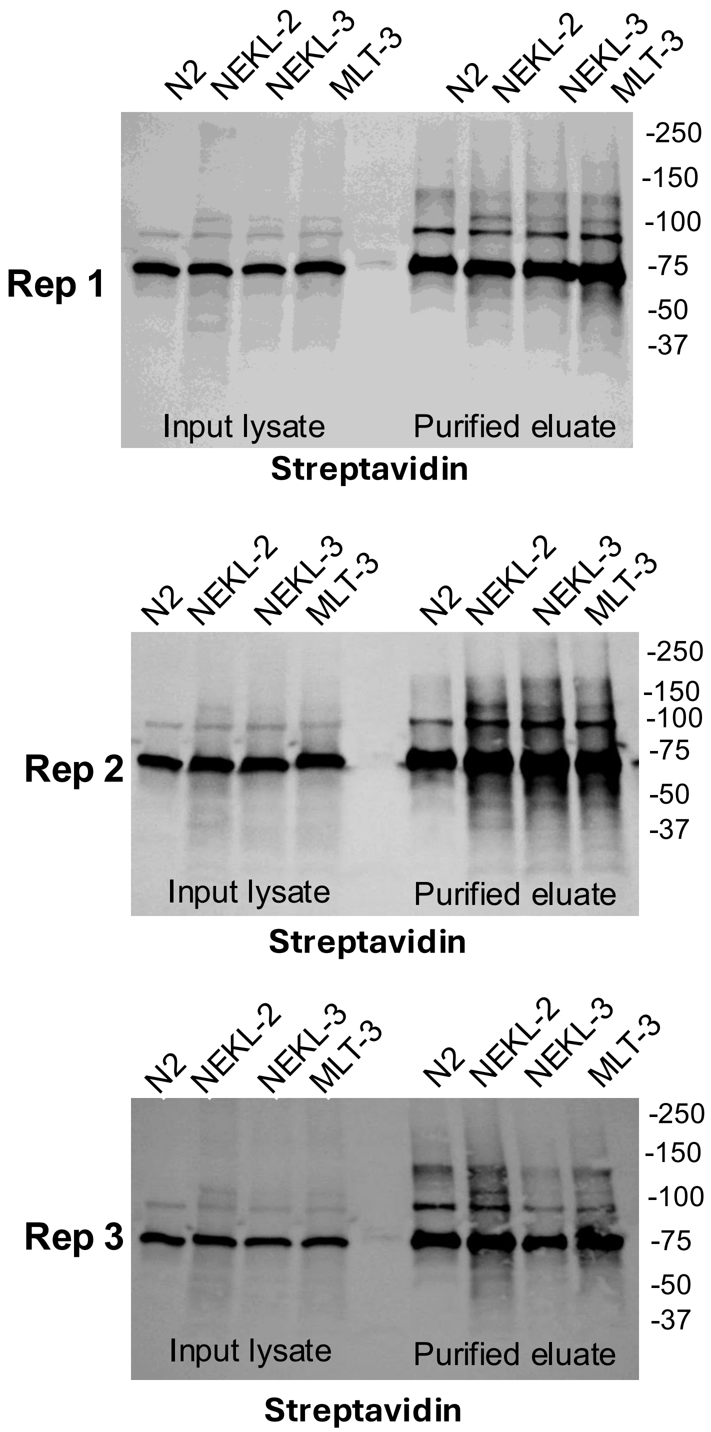

PYC-1a, 129.3 kDa; PYC-1b, 67.8 kDa; PCCA-1, 79.7 kDa; MCCC-1, 73.7 kDa; POD-2a, 230.6 kDa; POD-2b, 91.4 kD

MS Intensity: PYC-1 > PCCA-1 > MCCC-1 >> POD-2

CT-TurboID: NEKL-2 80.9 kD; NEKL-3 75.0 kD; MLT-2 115.3 kD; MLT-3 83.5 kD; MLT-4 109.9 kD

Experiment 2

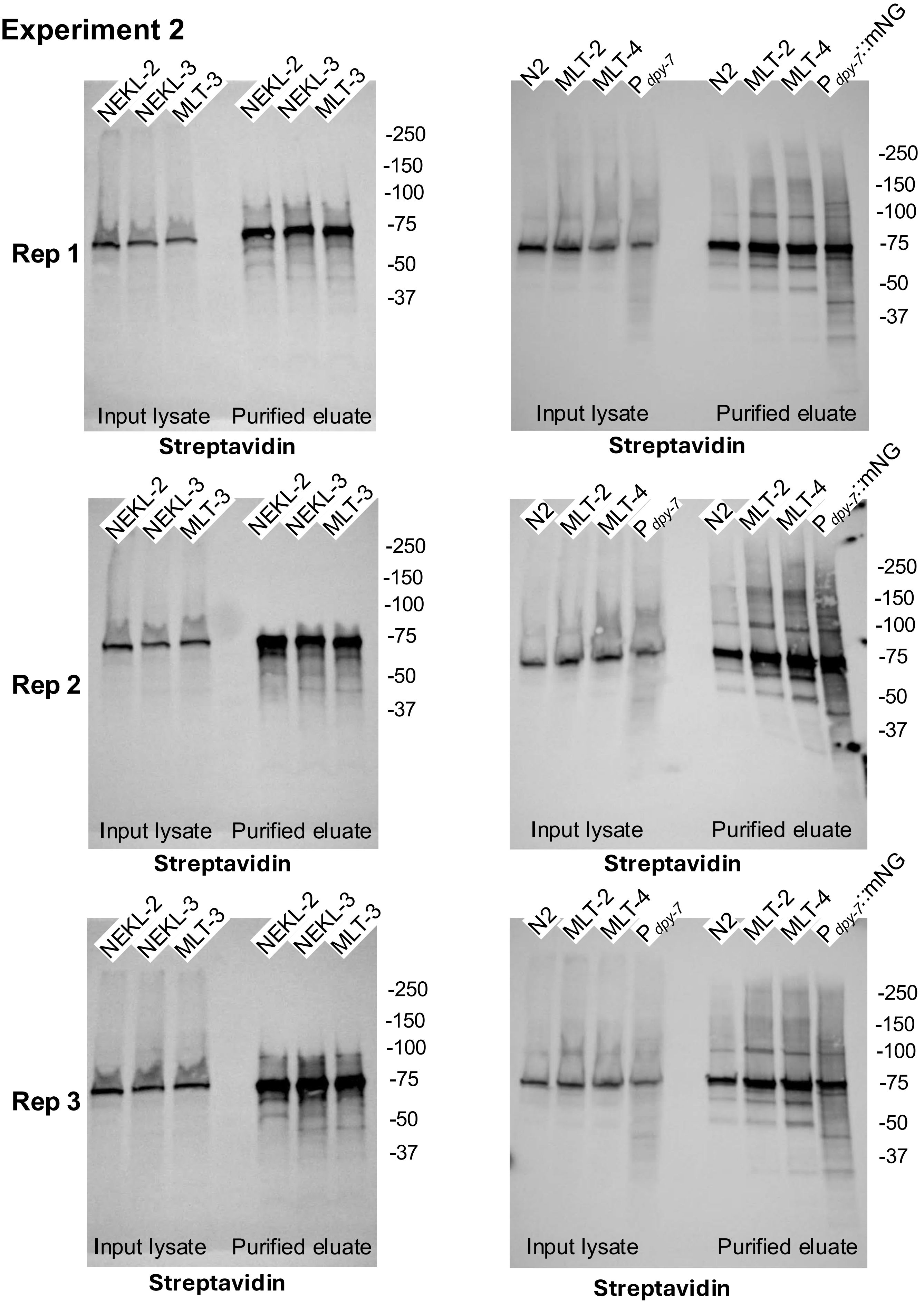

PYC-1a, 129.3 kDa; PYC-1b, 67.8 kDa; PCCA-1, 79.7 kDa; MCCC-1, 73.7 kDa; POD-2a, 230.6 kDa; POD-2b, 91.4 kD

Experiment 3

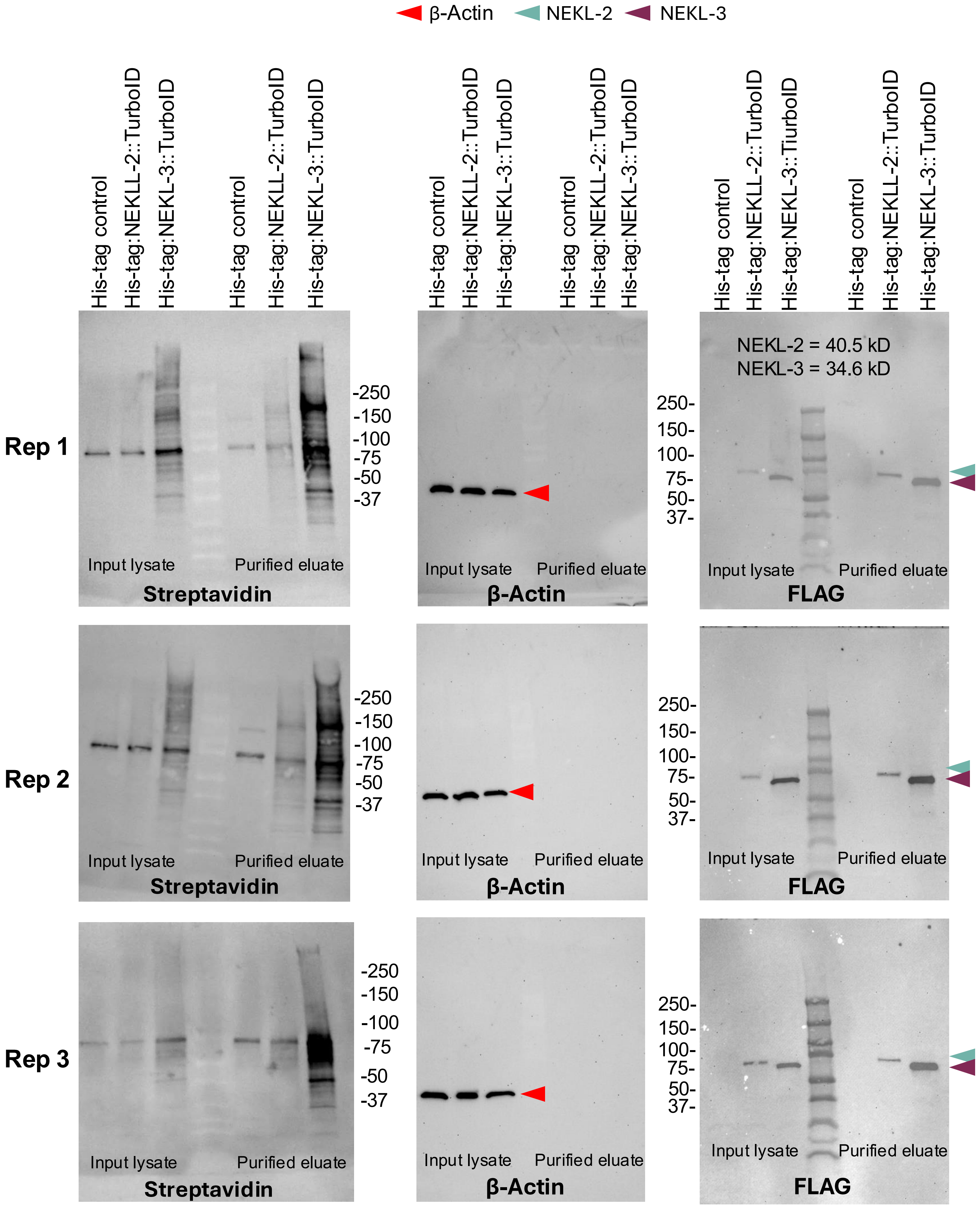

PYC-1a, 129.3 kDa; PYC-1b, 67.8 kDa; PCCA-1, 79.7 kDa; MCCC-1, 73.7 kDa; POD-2a, 230.6 kDa; POD-2b, 91.4 kD

CT-TurboID: NEKL-2 80.9 kDa; NEKL-3 75.0 kDa

Experiment 4

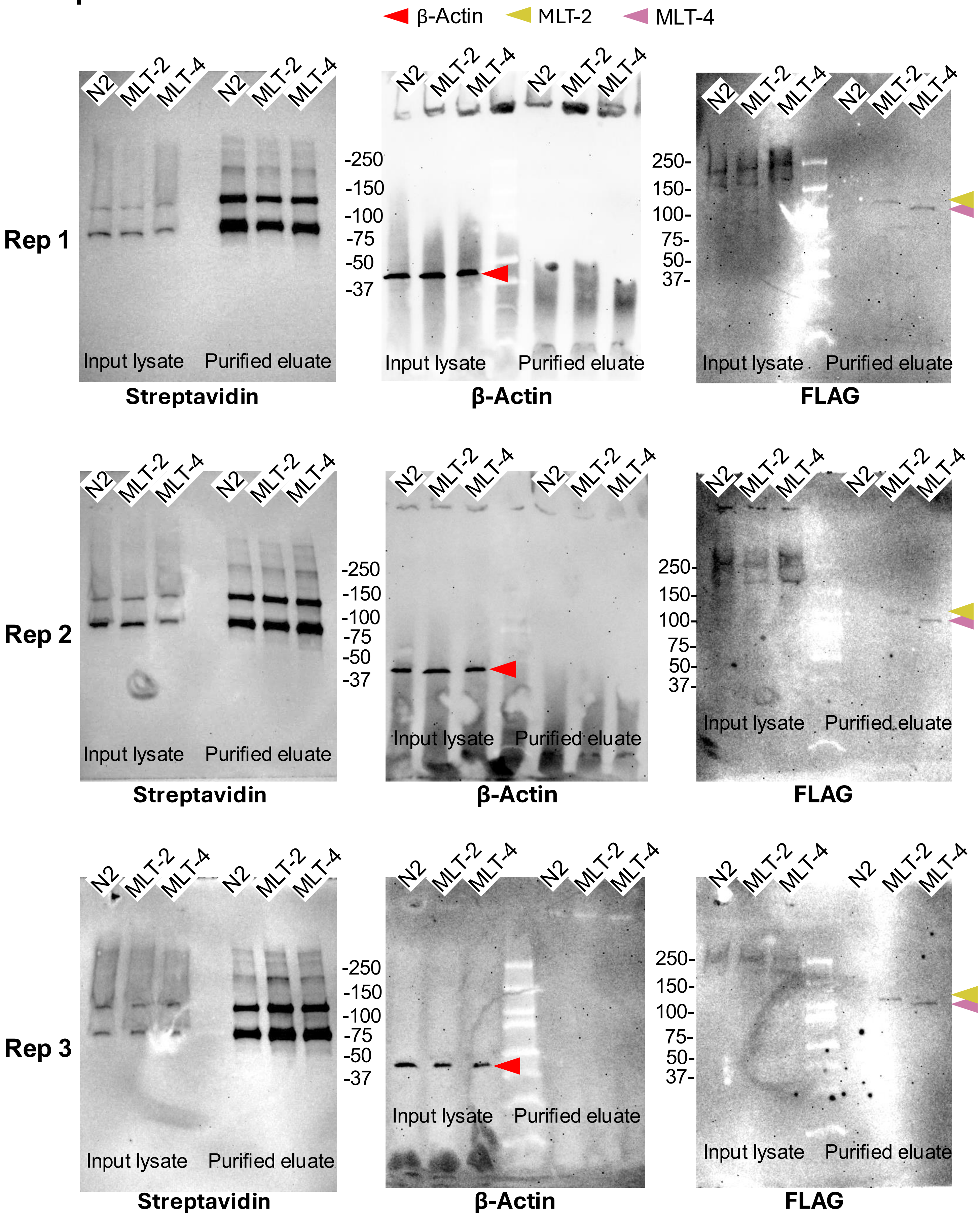

MLT-2 74.9 kDa; MLT-4 69.5 kDa

PYC-1a, 129.3 kDa; PYC-1b, 67.8 kDa; PCCA-1, 79.7 kDa; MCCC-1, 73.7 kDa; POD-2a, 230.6 kDa; POD-2b, 91.4 kD

CT-TurboID: MLT-2 115.3 kDa; MLT-4 109.9 kDa

Experiment 5

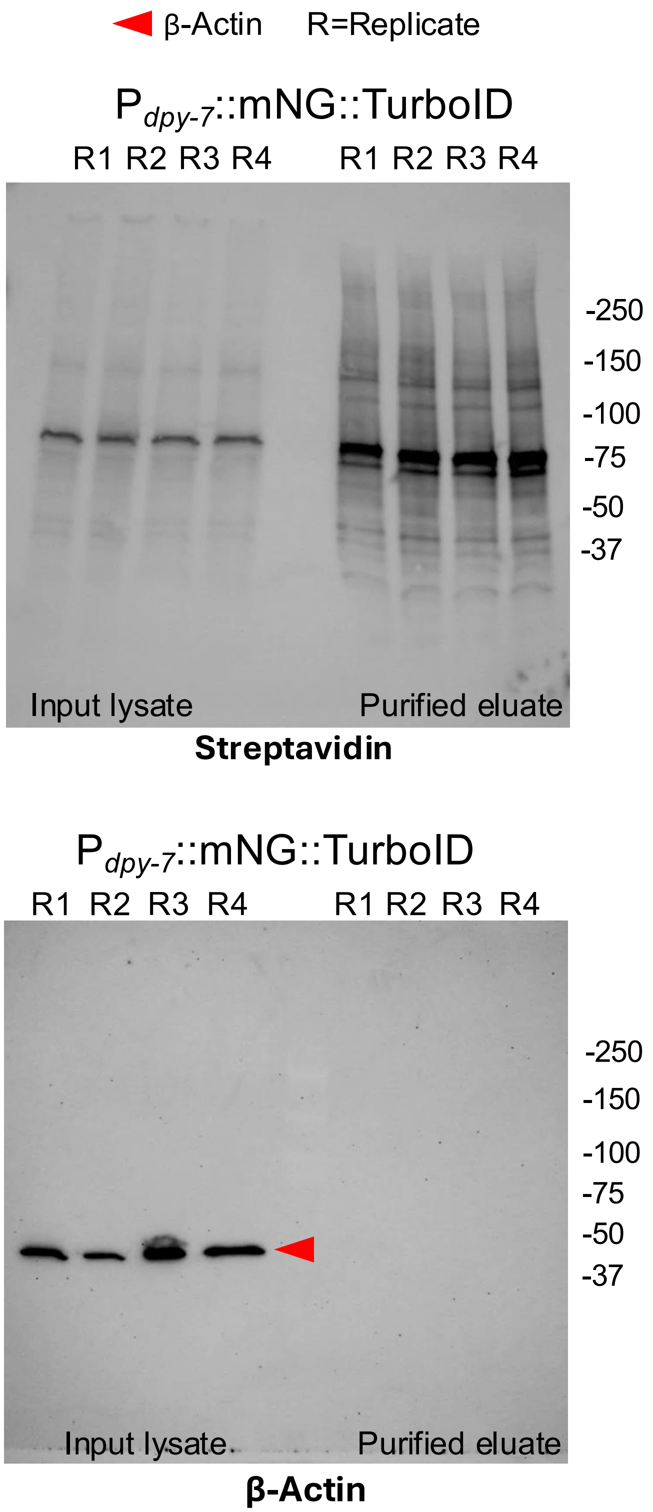

PYC-1a, 129.3 kDa; PYC-1b, 67.8 kDa; PCCA-1, 79.7 kDa; MCCC-1, 73.7 kDa; POD-2a, 230.6 kDa; POD-2b, 91.4 kD

Experiment 6

Rep 1

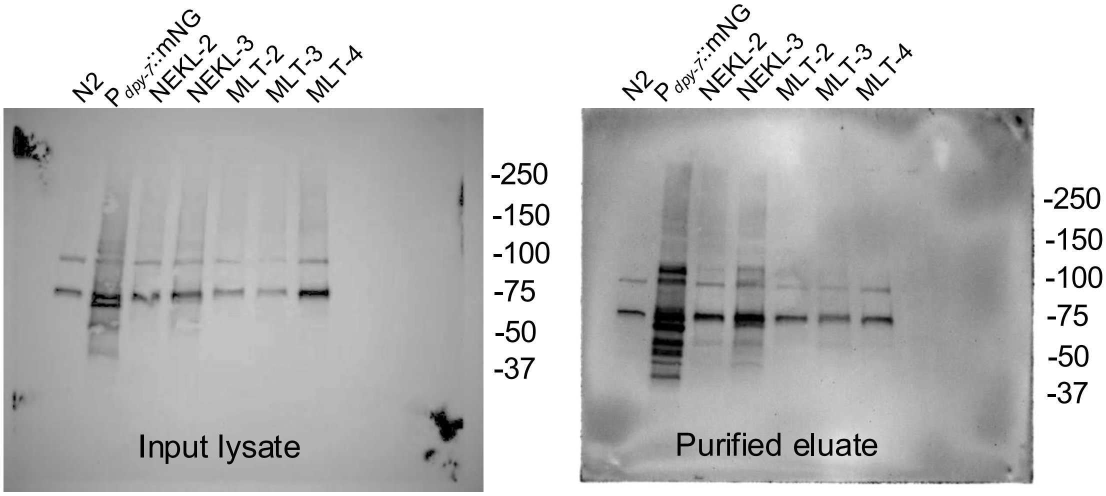

Streptavidin

Rep 2

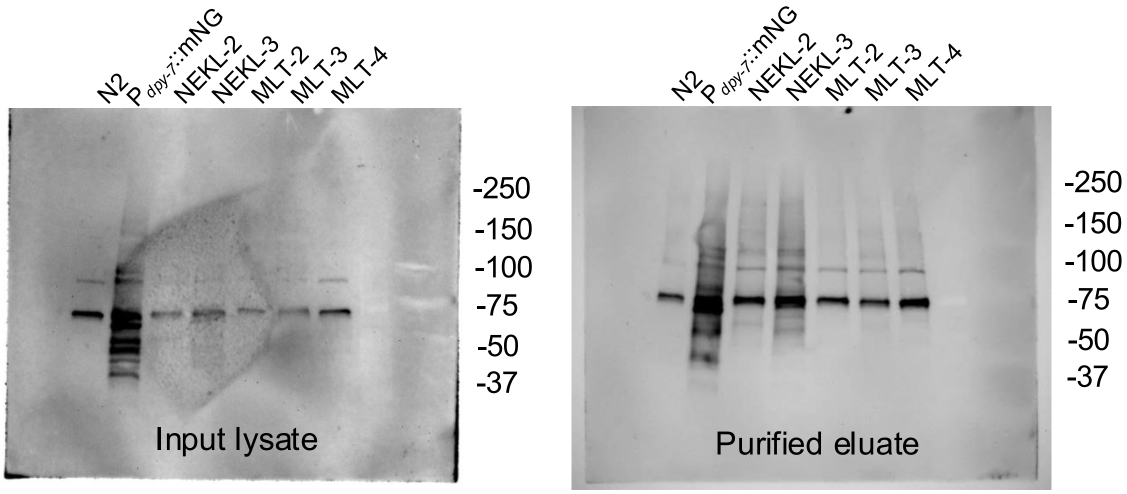

Streptavidin

Rep 3

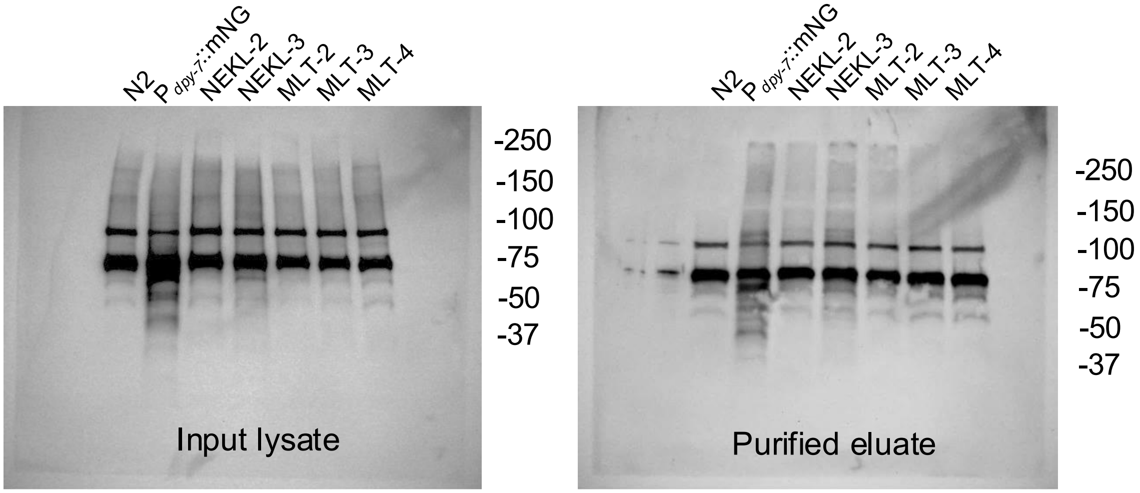

Streptavidin

PYC-1a, 129.3 kDa; PYC-1b, 67.8 kDa; PCCA-1, 79.7 kDa; MCCC-1, 73.7 kDa; POD-2a, 230.6 kDa; POD-2b, 91.4 kD

Experiment 6

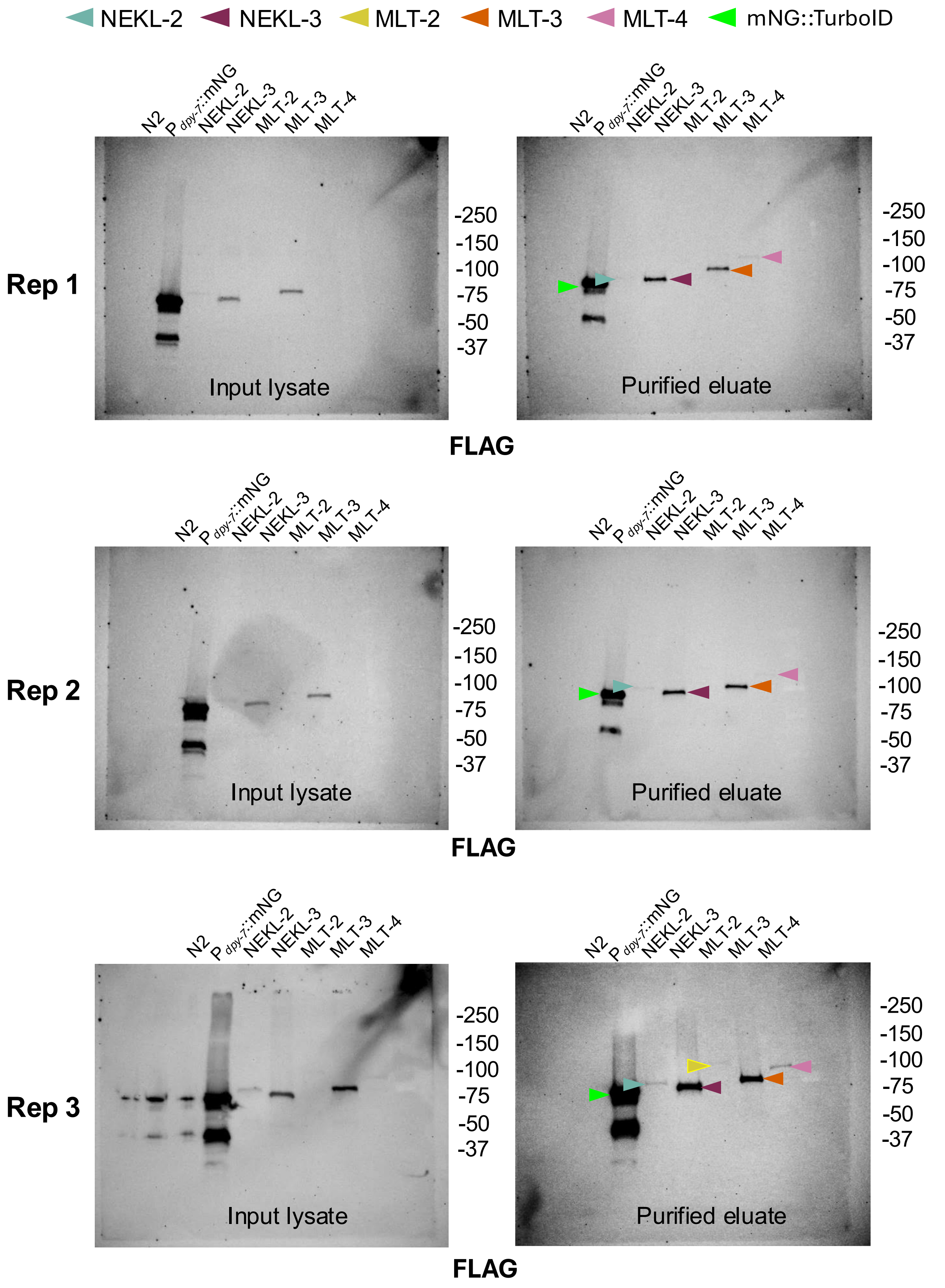

CT-TurboID: NEKL-2 80.9 kDa; NEKL-3 75.0 kDa; MLT-2 115.3 kDa; MLT-3 83.5 kDa; MLT-4 109.9 kDa
mNeonGreen::TurboID 68k Da

Experiment 6

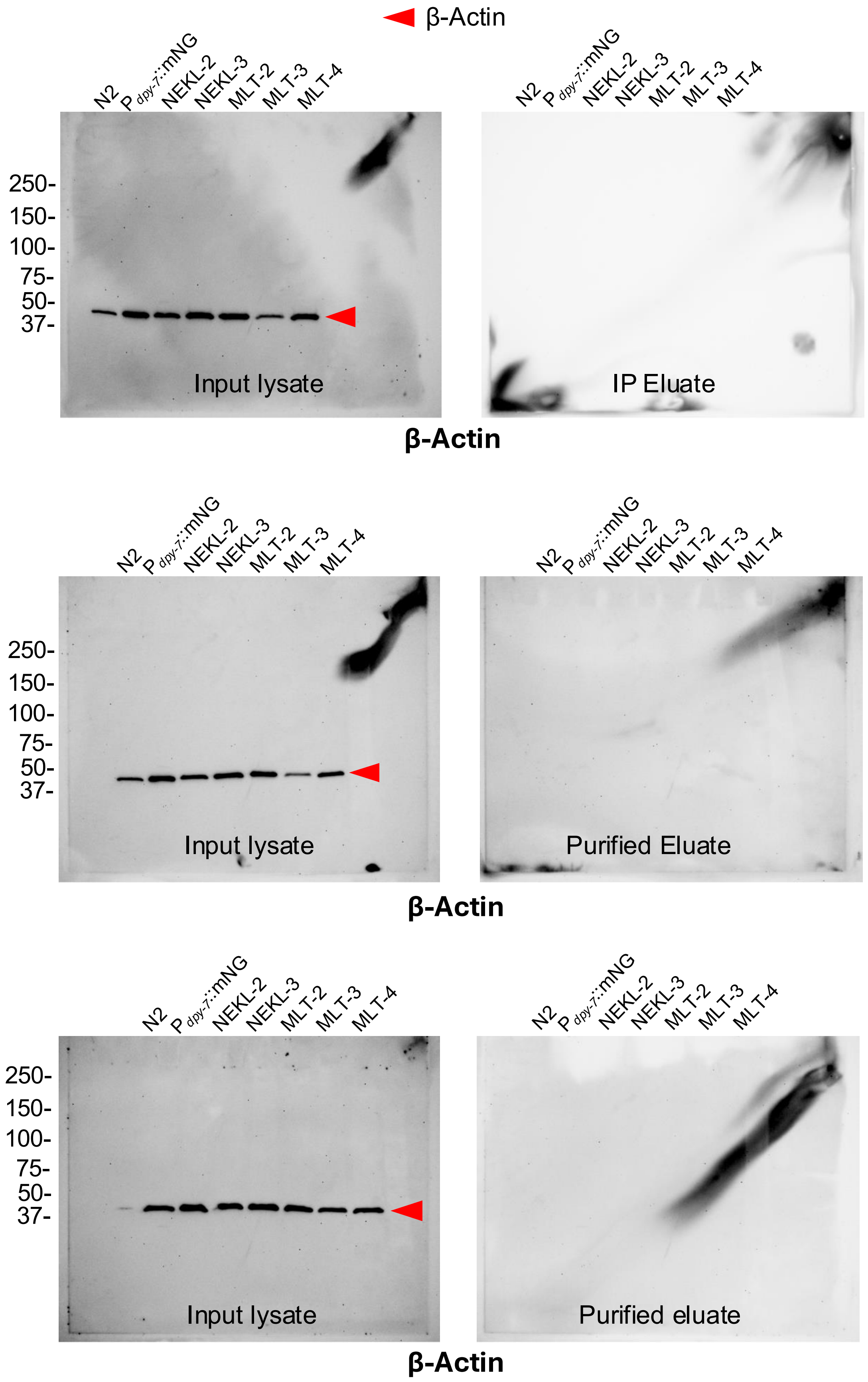

Experiment 7

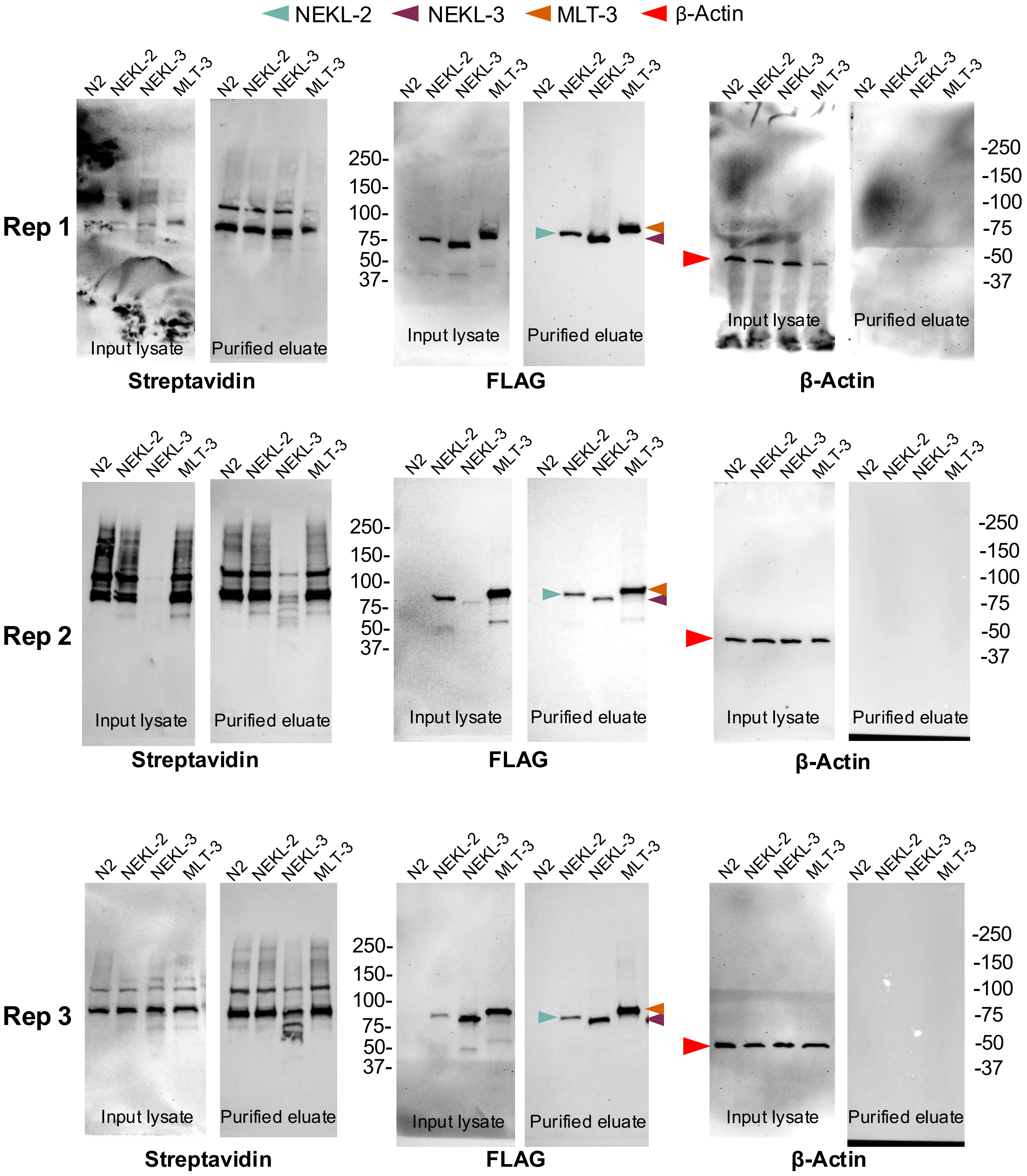

PYC-1a, 129.3 kDa; PYC-1b, 67.8 kDa; PCCA-1, 79.7 kDa; MCCC-1, 73.7 kDa; POD-2a, 230.6 kDa; POD-2b, 91.4 kD

CT-TurboID: NEKL-2 80.9 kDa; NEKL-3 75.0 kDa; MLT-3 83.5 kDa

Experiment 8

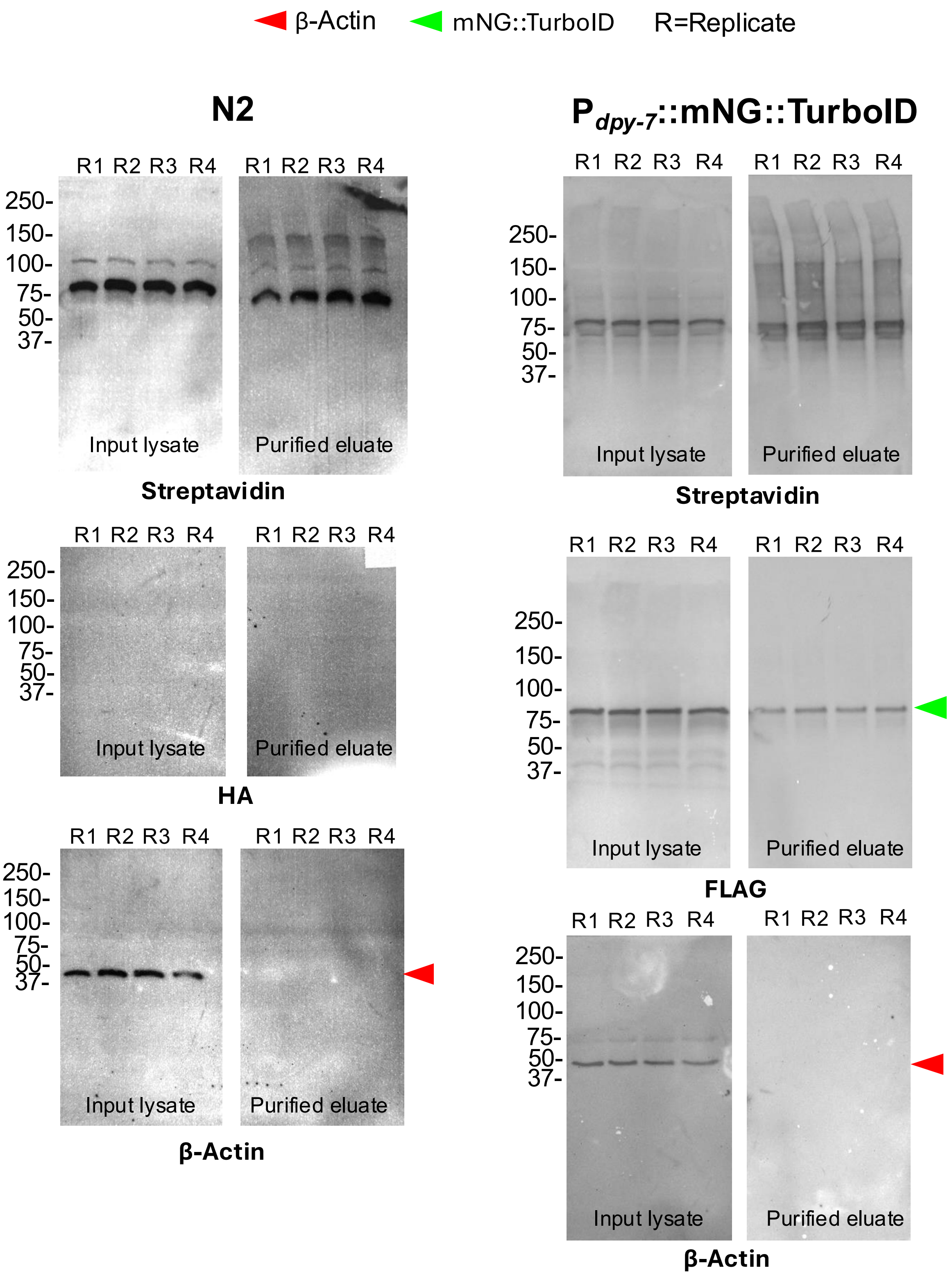

PYC-1a, 129.3 kDa; PYC-1b, 67.8 kDa; PCCA-1, 79.7 kDa; MCCC-1, 73.7 kDa; POD-2a, 230.6 kDa; POD-2b, 91.4 kD
mNeonGreen::TurboID 68k Da

Experiment 8

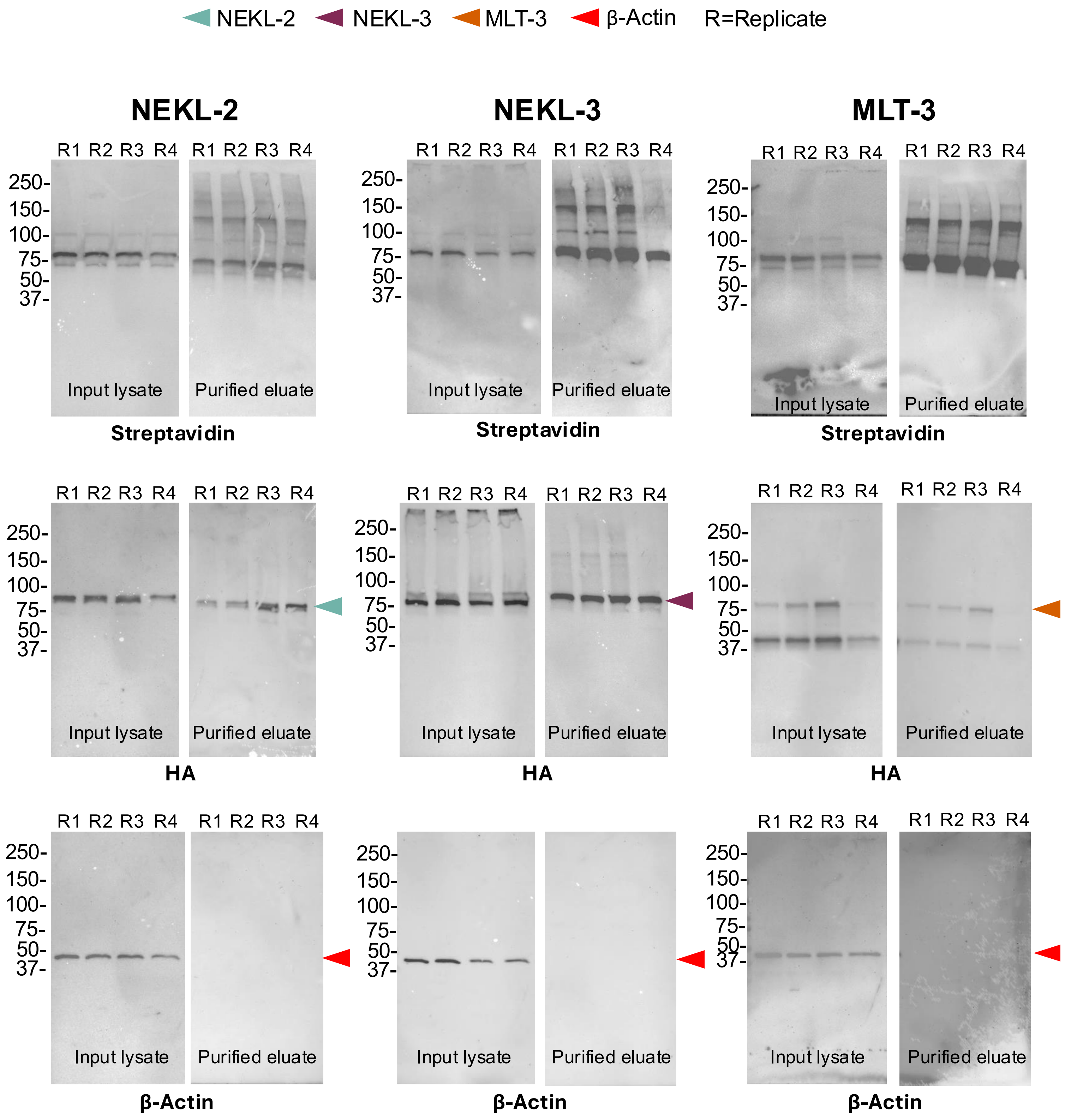

PYC-1a, 129.3 kDa; PYC-1b, 67.8 kDa; PCCA-1, 79.7 kDa; MCCC-1, 73.7 kDa; POD-2a, 230.6 kDa; POD-2b, 91.4 kD

NT-TurboID: NEKL-2 81.0 kDa; NEKL-3 75.1 kDa; MLT-3 83.4 kDa

Appendix Supplementary Methods S1

C-terminal fusions of TurboID

Linkers TurboID FLAG-3x **40.4 kDa**

Protein

GGGGSGSGGRGSGSGSGSGSGSGSGSGKDNTVPLKLIALLANGEFHSGEQLGETLGMSRAAI
NKHIQTLRDWGVDVFTVPGKGYSLPEPIPLLNAKQILGQLDGGSVAVLPVVDSTNQYLLDRIGE
LKSGDACIAEYQQAGRGSRGRKWFSPFGANLYLSMFWRLKRGPAAIGLGPVIGIVMAEALRKLG
ADKVRVKWPNDLYLQDRKLAGILVELAGITGDAAQIVIGAGINVAMRRVEESVVNQGWITLQEA
GINLDRNTLAATLIRELRAALELFEQEGLAPYLPRWEKLDNFINRPVKLIIGDKEIFGISRGID
KQGALLLEQDGVIKPWMGGEISLRSAEKAGGDYKDDDDKRDYKDDDDKRDYKDDDDK

DNA

GGAGGTGGTGGATCAGGCTCGGGAGGTCGAGGCTCAGGATCCGGTTCCGGCTCCGGCTCTGGTT
CCGGTTCGGGTTCCGGTTCTGGAAAGGATAACACCGTTCCACTTAAGCTTATCGCCCTTCTTGC
CAACGGAGAATTCCACTCTGGAGAGCAACTTGGAGAGACTCTTGGAATGTCCCGTGCTGCCATC
AACAAGCATATCCAAACCCTTCGTGATTGGGGAGTTGATGTTTTCACTGTTCCAGGAAAGgtaa
gtttaaacatatatatactaactaaccctgattatttaaattttcagGGATACTCCCTTCCAGA
GCCAATCCCACTTCTTAACGCCAAGCAAATCCTTGGACAACTTGATGGAGGATCCGTCGCTGTC
CTTCCAGTTGTTGATTCCACCAACCAATACCTTCTTGACCGTATCGGAGAGCTTAAGTCTGGAG
ACGCCTGCATCGCTGAGTACCAACAAGCTGGACGCGGATCTCGCGGACGCAAGTGGTTCTCCCC
ATTCGGAGCCAACCTTTACCTTTCTATGTTCTGGCGTCTTAAGCGTGGACCAGCTGCTATCGGA
CTTGGACCAGTTATCGGAATCGTTATGGCTGAGGCCCTTCGTAAGCTTGGAGCTGATAAGgtaa
gtttaaacagttcggtactaactaaccatacatatttaaattttcagGTTCGTGTTAAGTGGCC
AAACGATCTTTACCTTCAAGACCGTAAGCTTGCTGGAATCCTTGTCGAGCTTGCTGGAATCACC
GGAGACGCCGCTCAAATCGTTATCGGAGCTGGAATCAACGTTGCCATGCGTCGTGTTGAGGAGT
CTGTTGTTAACCAAGGATGGATCACTCTTCAAGAGGCTGGAATCAACCTTGATCGTAACACCCT
TGCTGCCACCCTTATCCGTGAGCTTCGTGCTGCCCTTGAGCTTTTCGAGCAAGAGGGACTTGCC
CCATACCTTCCACGCTGGGAGAAGCTTGACAACTTCATCAACCGCCCAGTTAAGCTTATCATCG
GAGATAAGGAAATCTTCGGAATCTCTCGCGGAATCGACAAGCAAGGAGCTCTTCTTCTTGAGCA
AGATGGAGTCATTAAGCCATGGATGGGAGGAGAGATTTCCCTTCGTTCCGCTGAGAAGGCCGGA
GGAGATTATAAAGACGATGACGATAAGCGTGACTACAAGGACGACGACGACAAGCGTGATTACA
AGGATGACGATGACAAG

Lower case in TurboID sequence indicates synthetic introns.

Final coding codon of NEKL-MLT bait proteins directly preceeds the N-terminal linker sequence.

Endogenous stop codon of NEKL-MLT bait proteins directly follows the C-terminal FLAG-3x sequence.

N-terminal fusions of TurboID

Linkers TurboID HA-3x **40.5 kDa**

Protein

PYDVPDYAGSYPYDVPDYAGSYPYDVPDYAGGGKDNTVPLKLIALLANGEFHSGEQLGETLGMS
RAAINKHIQTLRDWGVDVFTVPGKGYSLPEPIPLLNAKQILGQLDGGSVAVLPVVDSTNQYLLD
RIGELKSGDACIAEYQQAGRGSRGRKWFSPFGANLYLSMFWRLKRGPAAIGLGPVIGIVMAEAL
RKLGADKVRVKWPNDLYLQDRKLAGILVELAGITGDAAQIVIGAGINVAMRRVEESVVNQGWIT
LQEAGINLDRNTLAATLIRELRAALELFEQEGLAPYLPRWEKLDNFINRPVKLIIGDKEIFGIS
RGIDKQGALLLEQDGVIKPWMGGEISLRSAEKGGGGSGSGGRGSGSGSGSGSGSGSGSG

DNA

TACCCATATGACGTTCCAGACTACGCGGGTAGTTATCCGTACGACGTTCCGGATTACGCTGGAT
CTTACCCTTACGACGTACCTGACTACGCTGGAGGTGGTAAGGATAACACCGTTCCACTTAAGCT
TATCGCCCTTCTTGCCAACGGAGAATTCCACTCTGGAGAGCAACTTGGAGAGACTCTTGGAATG
TCCCGTGCTGCCATCAACAAGCATATCCAAACCCTTCGTGATTGGGGAGTTGATGTTTTCACTG
TTCCAGGAAAGgtaagtttaaacatatatatactaactaaccctgattatttaaattttcagGG
ATACTCCCTTCCAGAGCCAATCCCACTTCTTAACGCCAAGCAAATCCTTGGACAACTTGATGGA
GGATCCGTCGCTGTCCTTCCAGTTGTTGATTCCACCAACCAATACCTTCTTGACCGTATCGGAG
AGCTTAAGTCTGGAGACGCCTGCATCGCTGAGTACCAACAAGCTGGACGCGGATCTCGCGGACG
CAAGTGGTTCTCCCCATTCGGAGCCAACCTTTACCTTTCTATGTTCTGGCGTCTTAAGCGTGGA
CCAGCTGCTATCGGACTTGGACCAGTTATCGGAATCGTTATGGCTGAGGCCCTTCGTAAGCTTG
GAGCTGATAAGgtaagtttaaacagttcggtactaactaaccatacatatttaaattttcagGT
TCGTGTTAAGTGGCCAAACGATCTTTACCTTCAAGACCGTAAGCTTGCTGGAATCCTTGTCGAG
CTTGCTGGAATCACCGGAGACGCCGCTCAAATCGTTATCGGAGCTGGAATCAACGTTGCCATGC
GTCGTGTTGAGGAGTCTGTTGTTAACCAAGGATGGATCACTCTTCAAGAGGCTGGAATCAACCT
TGATCGTAACACCCTTGCTGCCACCCTTATCCGTGAGCTTCGTGCTGCCCTTGAGCTTTTCGAG
CAAGAGGGACTTGCCCCATACCTTCCACGCTGGGAGAAGCTTGACAACTTCATCAACCGCCCAG
TTAAGCTTATCATCGGAGATAAGGAAATCTTCGGAATCTCTCGCGGAATCGACAAGCAAGGAGC
TCTTCTTCTTGAGCAAGATGGAGTCATTAAGCCATGGATGGGAGGAGAGATTTCCCTTCGTTCC
GCTGAGAAGGGAGGTGGTGGATCAGGCTCGGGAGGTCGAGGCTCAGGATCCGGTTCCGGCTCCG
GCTCTGGTTCCGGTTCGGGTTCCGGTTCTGGA

Lower case in TurboID sequence indicates synthetic introns.

First codon of NEKL-MLT bait proteins (ATG) directly precedes the HA-3x tag sequence.

Second codon of NEKL-MLT bait proteins directly follows the C-terminal linker sequence.

Exp 1 FASP Methods – Orbitrap Exploris DIA

Protein samples were reduced, alkylated, and digested on-bead using filter-aided sample preparation [*Nature Methods* **6:** 359-62 (2009)] with sequencing grade modified porcine trypsin (Promega).

Tryptic peptides were trapped and eluted on 3.5um CSH C18 resin (Waters) (4mm x 75um) then separated by reverse phase XSelect CSH C18 2.5 um resin (Waters) on an in-line 150 x 0.075 mm column using an UltiMate 3000 RSLCnano system (Thermo). Peptides were eluted at a flow rate of 0.300uL/min using a 60 min gradient from 98% Buffer A:2% Buffer B to 95:5 at 2.0 minutes to 80:20 at 39.0 minutes to 60:40 at 48.0 minutes to 10:90 at 49.0 minutes and hold until 53.0 minutes and then equilibrated back to 98:2 at 53.1 minutes until 60 minutes. Eluted peptides were ionized by electrospray (2.4 kV) through a heated capillary (275°C) followed by data collection on an Orbitrap Exploris 480 mass spectrometer (Thermo Scientific).

Precursor spectra were acquired with a scan from 385-1015 Th at a resolution set to 60,000 with 100% AGC, max time of 50 msec, and an RF parameter at 40%. DIA was configured on the Orbitrap 480 to acquire 50 x 12 Th isolation windows at 15,000 resolution, normalized AGC target 500%, maximum injection time 40 ms). A second DIA was acquired in a staggered window (12 Th) pattern with optimized window placements.

Buffer A = 0.1% formic acid, 0.5% acetonitrile
Buffer B = 0.1% formic acid, 99.9% acetonitrile

Following data acquisition, data were searched using Spectronaut (Biognosys version 18.3) against the UniProt *Caenorhabditis elegans* database (August 2023) using the directDIA method with an identification precursor and protein q-value cutoff of 1%, generate decoys set to true, the protein inference workflow set to maxLFQ, inference algorithm set to IDPicker, quantity level set to MS2, cross-run normalization set to false, and the protein grouping quantification set to median peptide and precursor quantity. Protein MS2 intensity values were assessed for quality using ProteiNorm (Graw *et al.*). The data was normalized using VSN (Huber et al) and analyzed using proteoDA to perform statistical analysis using Linear Models for Microarray Data (limma) with empirical Bayes (eBayes) smoothing to the standard errors (Thurman et al, Ritchie et al). Proteins with an FDR adjusted p-value < 0.05 and a fold change > 2 were considered significant.

Acknowledgement:
IDeA National Resource for Quantitative Proteomics and NIH/NIGMS grant R24GM137786.

References:

1. Searle, B.C., Pino, L.K., Egertson, J.D. *et al.* Chromatogram libraries improve peptide detection and quantification by data independent acquisition mass spectrometry. *Nat Commun* **9,** 5128 (2018). https://doi.org/10.1038/s41467-018-07454-w

2. Graw S, Tang J, Zafar MK, Byrd AK, Bolden C, Peterson EC, and Byrum SD. proteiNorm – A user-friendly tool for normalization and analysis of TMT and label-free protein quantification. ACS Omega 2020 5(40), 25625-25633. DOI: 10.1021/acsomega.0c02564.

3. Thurman TJ, Washam CL, Alkam D, Bird JT, Gies A, Dhusia K, Robeson II MS, and Byrum SD. proteoDA: a package for quantitative proteomics. 2023. Journal of Open Source Software. DOI: 10.21105/joss.05184.

4. Bolstad, B., *preprocessCore: A collection of pre-processing functions.* 2019.

5. Ritchie, M.E., et al., *limma powers differential expression analyses for RNA-sequencing and microarray studies.* Nucleic Acids Res, 2015. **43**(7): p. e47.

6. Chawade, A., E. Alexandersson, and F. Levander, *Normalyzer: a tool for rapid evaluation of normalization methods for omics data sets.* J Proteome Res, 2014. **13**(6): p. 3114-20.

7. Huber, W., et al., *Variance stabilization applied to microarray data calibration and to the quantification of differential expression.* Bioinformatics, 2002. **18 Suppl 1**: p. S96-104.

8. Alhamdoosh, M., Ng, M., Wilson, N.J., Sheridan, J. M., Huynh, H., Wilson, M.J., Ritchie, M. E. 2016. Combining multiple tools outperforms individual methods in gene set enrichment analyses. bioRxiv

Exp 2 FASP Methods – Orbitrap Exploris DIA

Protein samples were reduced, alkylated, and digested on-bead using filter-aided sample preparation [*Nature Methods* **6:** 359-62 (2009)] with sequencing grade modified porcine trypsin (Promega). Tryptic peptides were then separated by reverse phase XSelect CSH C18 2.5 um resin (Waters) on an in-line 150 x 0.075 mm column using an UltiMate 3000 RSLCnano system (Thermo). Peptides were eluted using a 60 min gradient from 98:2 to 65:35 buffer A:B ratio. Eluted peptides were ionized by electrospray (2.2 kV) followed by mass spectrometric analysis on an Orbitrap Exploris 480 mass spectrometer (Thermo). To assemble a chromatogram library, six gas-phase fractions were acquired on the Orbitrap Exploris with 4 m/z DIA spectra (4 m/z precursor isolation windows at 30,000 resolution, normalized AGC target 100%, maximum inject time 66 ms) using a staggered window pattern from narrow mass ranges using optimized window placements. Precursor spectra were acquired after each DIA duty cycle, spanning the m/z range of the gas-phase fraction (i.e. 496-602 m/z, 60,000 resolution, normalized AGC target 100%, maximum injection time 50 ms). For wide-window acquisitions, the Orbitrap Exploris was configured to acquire a precursor scan (385-1015 m/z, 60,000 resolution, normalized AGC target 100%, maximum injection time 50 ms) followed by 50x 12 m/z DIA spectra (12 m/z precursor isolation windows at 15,000 resolution, normalized AGC target 100%, maximum injection time 33 ms) using a staggered window pattern with optimized window placements. Precursor spectra were acquired after each DIA duty cycle.

Buffer A = 0.1% formic acid, 0.5% acetonitrile
Buffer B = 0.1% formic acid, 99.9% acetonitrile

Following data acquisition, data were searched using Spectronaut (Biognosys version 18.5) against the UniProt *Caenorhabditis elegans* database (April 2023) using the directDIA method with an identification precursor and protein q-value cutoff of 1%, generate decoys set to true, the protein inference workflow set to maxLFQ, inference algorithm set to IDPicker, quantity level set to MS2, cross-run normalization set to false, and the protein grouping quantification set to median peptide and precursor quantity. Protein MS2 intensity values were assessed for quality using ProteiNorm (Graw *et al.*). The data was normalized using VSN (Huber et al) and analyzed using proteoDA to perform statistical analysis using Linear Models for Microarray Data (limma) with empirical Bayes (eBayes) smoothing to the standard errors (Thurman et al, Ritchie et al). Proteins with an FDR adjusted p-value < 0.05 and a fold change > 2 were considered significant.

Acknowledgement:
IDeA National Resource for Quantitative Proteomics and NIH/NIGMS grant R24GM137786.

References:

1. Searle, B.C., Pino, L.K., Egertson, J.D. *et al.* Chromatogram libraries improve peptide detection and quantification by data independent acquisition mass spectrometry. *Nat Commun* **9,** 5128 (2018). https://doi.org/10.1038/s41467-018-07454-w

2. Graw S, Tang J, Zafar MK, Byrd AK, Bolden C, Peterson EC, and Byrum SD. proteiNorm – A user-friendly tool for normalization and analysis of TMT and label-free protein quantification. ACS Omega 2020 5(40), 25625-25633. DOI: 10.1021/acsomega.0c02564.

3. Thurman TJ, Washam CL, Alkam D, Bird JT, Gies A, Dhusia K, Robeson II MS, and Byrum SD. proteoDA: a package for quantitative proteomics. 2023. Journal of Open Source Software. DOI: 10.21105/joss.05184.

4. Bolstad, B., *preprocessCore: A collection of pre-processing functions.* 2019.

5. Ritchie, M.E., et al., *limma powers differential expression analyses for RNA-sequencing and microarray studies.* Nucleic Acids Res, 2015. **43**(7): p. e47.

6. Chawade, A., E. Alexandersson, and F. Levander, *Normalyzer: a tool for rapid evaluation of normalization methods for omics data sets.* J Proteome Res, 2014. **13**(6): p. 3114-20.

7. Huber, W., et al., *Variance stabilization applied to microarray data calibration and to the quantification of differential expression.* Bioinformatics, 2002. **18 Suppl 1**: p. S96-104.

8. Alhamdoosh, M., Ng, M., Wilson, N.J., Sheridan, J. M., Huynh, H., Wilson, M.J., Ritchie, M. E. 2016. Combining multiple tools outperforms individual methods in gene set enrichment analyses. bioRxiv

Exp 3 FASP Methods – Orbitrap Exploris DIA

Protein samples were reduced, alkylated, and digested on-bead using filter-aided sample preparation [*Nature Methods* **6:** 359-62 (2009)] with sequencing grade modified porcine trypsin (Promega). Tryptic peptides were then separated by reverse phase XSelect CSH C18 2.5 um resin (Waters) on an in-line 150 x 0.075 mm column using an UltiMate 3000 RSLCnano system (Thermo). Peptides were eluted using a 60 min gradient from 98:2 to 65:35 buffer A:B ratio. Eluted peptides were ionized by electrospray (2.2 kV) followed by mass spectrometric analysis on an Orbitrap Exploris 480 mass spectrometer (Thermo). To assemble a chromatogram library, six gas-phase fractions were acquired on the Orbitrap Exploris with 4 m/z DIA spectra (4 m/z precursor isolation windows at 30,000 resolution, normalized AGC target 100%, maximum inject time 66 ms) using a staggered window pattern from narrow mass ranges using optimized window placements. Precursor spectra were acquired after each DIA duty cycle, spanning the m/z range of the gas-phase fraction (i.e. 496-602 m/z, 60,000 resolution, normalized AGC target 100%, maximum injection time 50 ms). For wide-window acquisitions, the Orbitrap Exploris was configured to acquire a precursor scan (385-1015 m/z, 60,000 resolution, normalized AGC target 100%, maximum injection time 50 ms) followed by 50x 12 m/z DIA spectra (12 m/z precursor isolation windows at 15,000 resolution, normalized AGC target 100%, maximum injection time 33 ms) using a staggered window pattern with optimized window placements. Precursor spectra were acquired after each DIA duty cycle.

Buffer A = 0.1% formic acid, 0.5% acetonitrile
Buffer B = 0.1% formic acid, 99.9% acetonitrile

Following data acquisition, data were searched using Spectronaut (Biognosys version 18.5) against the UniProt *Caenorhabditis elegans* database (September 2023) using the directDIA method with an identification precursor and protein q-value cutoff of 1%, generate decoys set to true, the protein inference workflow set to maxLFQ, inference algorithm set to IDPicker, quantity level set to MS2, cross-run normalization set to false, and the protein grouping quantification set to median peptide and precursor quantity. Protein MS2 intensity values were assessed for quality using ProteiNorm (Graw *et al.*). The data was normalized using VSN (Huber et al) and analyzed using proteoDA to perform statistical analysis using Linear Models for Microarray Data (limma) with empirical Bayes (eBayes) smoothing to the standard errors (Thurman et al, Ritchie et al). Proteins with an FDR adjusted p-value < 0.05 and a fold change > 2 were considered significant.

Acknowledgement:
IDeA National Resource for Quantitative Proteomics and NIH/NIGMS grant R24GM137786.

References:

1. Searle, B.C., Pino, L.K., Egertson, J.D. *et al.* Chromatogram libraries improve peptide detection and quantification by data independent acquisition mass spectrometry. *Nat Commun* **9,** 5128 (2018). https://doi.org/10.1038/s41467-018-07454-w

2. Graw S, Tang J, Zafar MK, Byrd AK, Bolden C, Peterson EC, and Byrum SD. proteiNorm – A user-friendly tool for normalization and analysis of TMT and label-free protein quantification. ACS Omega 2020 5(40), 25625-25633. DOI: 10.1021/acsomega.0c02564.

3. Thurman TJ, Washam CL, Alkam D, Bird JT, Gies A, Dhusia K, Robeson II MS, and Byrum SD. proteoDA: a package for quantitative proteomics. 2023. Journal of Open Source Software. DOI: 10.21105/joss.05184.

4. Bolstad, B., *preprocessCore: A collection of pre-processing functions.* 2019.

5. Ritchie, M.E., et al., *limma powers differential expression analyses for RNA-sequencing and microarray studies.* Nucleic Acids Res, 2015. **43**(7): p. e47.

6. Chawade, A., E. Alexandersson, and F. Levander, *Normalyzer: a tool for rapid evaluation of normalization methods for omics data sets.* J Proteome Res, 2014. **13**(6): p. 3114-20.

7. Huber, W., et al., *Variance stabilization applied to microarray data calibration and to the quantification of differential expression.* Bioinformatics, 2002. **18 Suppl 1**: p. S96-104.

8. Alhamdoosh, M., Ng, M., Wilson, N.J., Sheridan, J. M., Huynh, H., Wilson, M.J., Ritchie, M. E. 2016. Combining multiple tools outperforms individual methods in gene set enrichment analyses. bioRxiv

Exp 4 FASP Methods – Orbitrap Exploris DIA

Protein samples were reduced, alkylated, and digested on-bead using filter-aided sample preparation [*Nature Methods* **6:** 359-62 (2009)] with sequencing grade modified porcine trypsin (Promega). Tryptic peptides were then separated by reverse phase XSelect CSH C18 2.5 um resin (Waters) on an in-line 150 x 0.075 mm column using an UltiMate 3000 RSLCnano system (Thermo). Peptides were eluted using a 60 min gradient from 98:2 to 65:35 buffer A:B ratio. Eluted peptides were ionized by electrospray (2.2 kV) followed by mass spectrometric analysis on an Orbitrap Exploris 480 mass spectrometer (Thermo). To assemble a chromatogram library, six gas-phase fractions were acquired on the Orbitrap Exploris with 4 m/z DIA spectra (4 m/z precursor isolation windows at 30,000 resolution, normalized AGC target 100%, maximum inject time 66 ms) using a staggered window pattern from narrow mass ranges using optimized window placements. Precursor spectra were acquired after each DIA duty cycle, spanning the m/z range of the gas-phase fraction (i.e. 496-602 m/z, 60,000 resolution, normalized AGC target 100%, maximum injection time 50 ms). For wide-window acquisitions, the Orbitrap Exploris was configured to acquire a precursor scan (385-1015 m/z, 60,000 resolution, normalized AGC target 100%, maximum injection time 50 ms) followed by 50x 12 m/z DIA spectra (12 m/z precursor isolation windows at 15,000 resolution, normalized AGC target 100%, maximum injection time 33 ms) using a staggered window pattern with optimized window placements. Precursor spectra were acquired after each DIA duty cycle.

Buffer A = 0.1% formic acid, 0.5% acetonitrile
Buffer B = 0.1% formic acid, 99.9% acetonitrile

Following data acquisition, data were searched using Spectronaut (Biognosys version 18.5) against the UniProt *Caenorhabditis elegans* database (September 2023) using the directDIA method with an identification precursor and protein q-value cutoff of 1%, generate decoys set to true, the protein inference workflow set to maxLFQ, inference algorithm set to IDPicker, quantity level set to MS2, cross-run normalization set to false, and the protein grouping quantification set to median peptide and precursor quantity. Protein MS2 intensity values were assessed for quality using ProteiNorm (Graw *et al.*). The data was normalized using VSN (Huber et al) and analyzed using proteoDA to perform statistical analysis using Linear Models for Microarray Data (limma) with empirical Bayes (eBayes) smoothing to the standard errors (Thurman et al, Ritchie et al). Proteins with an FDR adjusted p-value < 0.05 and a fold change > 2 were considered significant.

Acknowledgement:
IDeA National Resource for Quantitative Proteomics and NIH/NIGMS grant R24GM137786.

References:

1. Searle, B.C., Pino, L.K., Egertson, J.D. *et al.* Chromatogram libraries improve peptide detection and quantification by data independent acquisition mass spectrometry. *Nat Commun* **9,** 5128 (2018). https://doi.org/10.1038/s41467-018-07454-w

2. Graw S, Tang J, Zafar MK, Byrd AK, Bolden C, Peterson EC, and Byrum SD. proteiNorm – A user-friendly tool for normalization and analysis of TMT and label-free protein quantification. ACS Omega 2020 5(40), 25625-25633. DOI: 10.1021/acsomega.0c02564.

3. Thurman TJ, Washam CL, Alkam D, Bird JT, Gies A, Dhusia K, Robeson II MS, and Byrum SD. proteoDA: a package for quantitative proteomics. 2023. Journal of Open Source Software. DOI: 10.21105/joss.05184.

4. Bolstad, B., *preprocessCore: A collection of pre-processing functions.* 2019.

5. Ritchie, M.E., et al., *limma powers differential expression analyses for RNA-sequencing and microarray studies.* Nucleic Acids Res, 2015. **43**(7): p. e47.

6. Chawade, A., E. Alexandersson, and F. Levander, *Normalyzer: a tool for rapid evaluation of normalization methods for omics data sets.* J Proteome Res, 2014. **13**(6): p. 3114-20.

7. Huber, W., et al., *Variance stabilization applied to microarray data calibration and to the quantification of differential expression.* Bioinformatics, 2002. **18 Suppl 1**: p. S96-104.

8. Alhamdoosh, M., Ng, M., Wilson, N.J., Sheridan, J. M., Huynh, H., Wilson, M.J., Ritchie, M. E. 2016. Combining multiple tools outperforms individual methods in gene set enrichment analyses. bioRxiv

Exp 5 Methods – FASP - Eclipse -DDA:

Protein samples were reduced, alkylated, and digested on-bead using filter-aided sample preparation [*Nature Methods* **6:** 359-62 (2009)] with sequencing grade modified porcine trypsin (Promega).
Tryptic peptides were separated by reverse phase XSelect CSH C18 2.5 um resin (Waters) on an in-line 150 x 0.075 mm column using an UltiMate 3000 RSLCnano system (Thermo). Peptides were eluted using a 90 min gradient from 98:2 to 65:35 buffer A:B ratio. Eluted peptides were ionized by electrospray (2.4 kV) followed by mass spectrometric analysis on an Orbitrap Eclipse Tribrid mass spectrometer (Thermo). MS data were acquired using the FTMS analyzer in profile mode at a resolution of 120,000 over a range of 375 to 1200 m/z. Following HCD activation, MS/MS data were acquired using the ion trap analyzer in centroid mode and normal mass range with a normalized collision energy of 30%. Proteins were identified by database search using MaxQuant (Max Planck Institute) label-free quantification with a parent ion tolerance of 2.5 ppm and a fragment ion tolerance of 0.5 Da. Scaffold Q+S (Proteome Software) was used to verify MS/MS based peptide and protein identifications. Protein identifications were accepted if they could be established with less than 1.0% false discovery and contained at least 2 identified peptides. Protein probabilities were assigned by the Protein Prophet algorithm [*Anal. Chem.* **75:** 4646-58 (2003)].

Buffer A = 0.1% formic acid, 0.5% acetonitrile
Buffer B = 0.1% formic acid, 99.9% acetonitrile

Data Analysis

Proteins were identified and quantified by searching the UniprotKB database restricted to *Caenorhabditis elegans* (February 2024) using MaxQuant (version 2.2.0.0, Max Planck Institute). The database search parameters included selecting the MS1 reporter type, trypsin digestion with up to two missed cleavages, fixed modifications for carbamidomethyl of cysteine, variable modifications for oxidation on methionine and acetyl on N-terminus, the precursor ion tolerance of 5 ppm for the first search and 3 ppm for the main search, and label-free quantitation with iBAQ. Peptide and protein identifications were accepted using the 1.0% false discovery rate identification threshold. Protein probabilities were assigned by the Protein Prophet algorithm (Nesvizhskii et al 2003).

MaxQuant iBAQ intensities for each sample were assessed for quality and differential abundance using proteoDA (Thurman et al. 2023; Graw et al. 2020). The data was normalized using VSN (Huber et al. 2002) and statistical analysis was performed using linear models for microarray data (limma) with empirical Bayes (eBayes) smoothing to the standard errors (Ritchie et al.2015). Proteins with an FDR adjusted p-value < 0.05 and a fold change > 2 were considered significant.

Acknowledgement:
IDeA National Resource for Quantitative Proteomics and NIH/NIGMS grant R24GM137786.

References:

1. Nesvizhskii AI, Keller A, Kolker E, Aebersold R. A statistical model for identifying proteins by tandem mass spectrometry. Anal Chem. 2003 Sep 1;75(17):4646-58. doi: 10.1021/ac0341261. PMID: 14632076.

2. Thurman et al. proteoDA: a package for quantitative proteomics. Journal of Open Source Software 2023 8(85), 5184, https://doi.org/10.21105/joss.05184.

3. Graw S, Tang J, Zafar MK, Byrd AK, Bolden C, Peterson EC, and Byrum SD. proteiNorm – A user-friendly tool for normalization and analysis of TMT and label-free protein quantification. ACS Omega 2020 5(40), 25625-25633. DOI: 10.1021/acsomega.0c02564.

4. Bolstad, B., *preprocessCore: A collection of pre-processing functions.* 2019.

5. Ritchie, M.E., et al., *limma powers differential expression analyses for RNA-sequencing and microarray studies.* Nucleic Acids Res, 2015. **43**(7): p. e47.

6. Chawade, A., E. Alexandersson, and F. Levander, *Normalyzer: a tool for rapid evaluation of normalization methods for omics data sets.* J Proteome Res, 2014. **13**(6): p. 3114-20.

5. Huber, W., et al., *Variance stabilization applied to microarray data calibration and to the quantification of differential expression.* Bioinformatics, 2002. **18 Suppl 1**: p. S96-104.

6. Alhamdoosh, M., Ng, M., Wilson, N.J., Sheridan, J. M., Huynh, H., Wilson, M.J., Ritchie, M. E. 2016. Combining multiple tools outperforms individual methods in gene set enrichment analyses. bioRxiv

Exp 6 FASP Methods – Orbitrap Exploris DIA

Protein samples were reduced, alkylated, and digested on-bead using filter-aided sample preparation [*Nature Methods* **6:** 359-62 (2009)] with sequencing grade modified porcine trypsin (Promega).

Tryptic peptides were trapped and eluted on 3.5um CSH C18 resin (Waters) (4mm x 75um) then separated by reverse phase XSelect CSH C18 2.5 um resin (Waters) on an in-line 150 x 0.075 mm column using an UltiMate 3000 RSLCnano system (Thermo). Peptides were eluted at a flow rate of 0.300uL/min using a 60 min gradient from 98% Buffer A:2% Buffer B to 95:5 at 2.0 minutes to 80:20 at 39.0 minutes to 60:40 at 48.0 minutes to 10:90 at 49.0 minutes and hold until 53.0 minutes and then equilibrated back to 98:2 at 53.1 minutes until 60 minutes. Eluted peptides were ionized by electrospray (2.4 kV) through a heated capillary (275°C) followed by data collection on an Orbitrap Exploris 480 mass spectrometer (Thermo Scientific).

Precursor spectra were acquired with a scan from 385-1015 Th at a resolution set to 60,000 with 100% AGC, max time of 50 msec, and an RF parameter at 40%. DIA was configured on the Orbitrap 480 to acquire 50 x 12 Th isolation windows at 15,000 resolution, normalized AGC target 500%, maximum injection time 40 ms). A second DIA was acquired in a staggered window (12 Th) pattern with optimized window placements.

Buffer A = 0.1% formic acid, 0.5% acetonitrile
Buffer B = 0.1% formic acid, 99.9% acetonitrile

Following data acquisition, data were searched using Spectronaut (Biognosys version 19.1) against the UniProt *Caenorhabditis elegans* database (April 2024) using the directDIA method with an identification precursor and protein q-value cutoff of 1%, generate decoys set to true, the protein inference workflow set to maxLFQ, inference algorithm set to IDPicker, quantity level set to MS2, cross-run normalization set to false, and the protein grouping quantification set to median peptide and precursor quantity. Protein MS2 intensity values were assessed for quality using ProteiNorm (Graw *et al.*). The data was normalized using VSN (Huber et al) and analyzed using proteoDA to perform statistical analysis using Linear Models for Microarray Data (limma) with empirical Bayes (eBayes) smoothing to the standard errors (Thurman et al, Ritchie et al). Proteins with an FDR adjusted p-value < 0.05 and a fold change > 2 were considered significant.

Acknowledgement:
IDeA National Resource for Quantitative Proteomics and NIH/NIGMS grant R24GM137786.

References:

1. Searle, B.C., Pino, L.K., Egertson, J.D. *et al.* Chromatogram libraries improve peptide detection and quantification by data independent acquisition mass spectrometry. *Nat Commun* **9,** 5128 (2018). https://doi.org/10.1038/s41467-018-07454-w

2. Graw S, Tang J, Zafar MK, Byrd AK, Bolden C, Peterson EC, and Byrum SD. proteiNorm – A user-friendly tool for normalization and analysis of TMT and label-free protein quantification. ACS Omega 2020 5(40), 25625-25633. DOI: 10.1021/acsomega.0c02564.

3. Thurman TJ, Washam CL, Alkam D, Bird JT, Gies A, Dhusia K, Robeson II MS, and Byrum SD. proteoDA: a package for quantitative proteomics. 2023. Journal of Open Source Software. DOI: 10.21105/joss.05184.

4. Bolstad, B., *preprocessCore: A collection of pre-processing functions.* 2019.

5. Ritchie, M.E., et al., *limma powers differential expression analyses for RNA-sequencing and microarray studies.* Nucleic Acids Res, 2015. **43**(7): p. e47.

6. Chawade, A., E. Alexandersson, and F. Levander, *Normalyzer: a tool for rapid evaluation of normalization methods for omics data sets.* J Proteome Res, 2014. **13**(6): p. 3114-20.

7. Huber, W., et al., *Variance stabilization applied to microarray data calibration and to the quantification of differential expression.* Bioinformatics, 2002. **18 Suppl 1**: p. S96-104.

8. Alhamdoosh, M., Ng, M., Wilson, N.J., Sheridan, J. M., Huynh, H., Wilson, M.J., Ritchie, M. E. 2016. Combining multiple tools outperforms individual methods in gene set enrichment analyses. bioRxiv

Exp 7 FASP Methods – Orbitrap Exploris DIA

Protein samples were reduced, alkylated, and digested on-bead using filter-aided sample preparation [*Nature Methods* **6:** 359-62 (2009)] with sequencing grade modified porcine trypsin (Promega).

Tryptic peptides were trapped and eluted on 3.5um CSH C18 resin (Waters) (4mm x 75um) then separated by reverse phase XSelect CSH C18 2.5 um resin (Waters) on an in-line 150 x 0.075 mm column using an UltiMate 3000 RSLCnano system (Thermo). Peptides were eluted at a flow rate of 0.300uL/min using a 60 min gradient from 98% Buffer A:2% Buffer B to 95:5 at 2.0 minutes to 80:20 at 39.0 minutes to 60:40 at 48.0 minutes to 10:90 at 49.0 minutes and hold until 53.0 minutes and then equilibrated back to 98:2 at 53.1 minutes until 60 minutes. Eluted peptides were ionized by electrospray (2.4 kV) through a heated capillary (275°C) followed by data collection on an Orbitrap Exploris 480 mass spectrometer (Thermo Scientific).

Precursor spectra were acquired with a scan from 385-1015 Th at a resolution set to 60,000 with 100% AGC, max time of 50 msec, and an RF parameter at 40%. DIA was configured on the Orbitrap 480 to acquire 50 x 12 Th isolation windows at 15,000 resolution, normalized AGC target 500%, maximum injection time 40 ms). A second DIA was acquired in a staggered window (12 Th) pattern with optimized window placements.

Buffer A = 0.1% formic acid, 0.5% acetonitrile
Buffer B = 0.1% formic acid, 99.9% acetonitrile

Following data acquisition, data were searched using Spectronaut (Biognosys version 19.1) against the UniProt *Caenorhabditis elegans* database (April 2024) using the directDIA method with an identification precursor and protein q-value cutoff of 1%, generate decoys set to true, the protein inference workflow set to maxLFQ, inference algorithm set to IDPicker, quantity level set to MS2, cross-run normalization set to false, and the protein grouping quantification set to median peptide and precursor quantity. Protein MS2 intensity values were assessed for quality using ProteiNorm (Graw *et al.*). The data was normalized using VSN (Huber et al) and analyzed using proteoDA to perform statistical analysis using Linear Models for Microarray Data (limma) with empirical Bayes (eBayes) smoothing to the standard errors (Thurman et al, Ritchie et al). Proteins with an FDR adjusted p-value < 0.05 and a fold change > 2 were considered significant.

Acknowledgement:
IDeA National Resource for Quantitative Proteomics and NIH/NIGMS grant R24GM137786.

References:

1. Searle, B.C., Pino, L.K., Egertson, J.D. *et al.* Chromatogram libraries improve peptide detection and quantification by data independent acquisition mass spectrometry. *Nat Commun* **9,** 5128 (2018). https://doi.org/10.1038/s41467-018-07454-w

2. Graw S, Tang J, Zafar MK, Byrd AK, Bolden C, Peterson EC, and Byrum SD. proteiNorm – A user-friendly tool for normalization and analysis of TMT and label-free protein quantification. ACS Omega 2020 5(40), 25625-25633. DOI: 10.1021/acsomega.0c02564.

3. Thurman TJ, Washam CL, Alkam D, Bird JT, Gies A, Dhusia K, Robeson II MS, and Byrum SD. proteoDA: a package for quantitative proteomics. 2023. Journal of Open Source Software. DOI: 10.21105/joss.05184.

4. Bolstad, B., *preprocessCore: A collection of pre-processing functions.* 2019.

5. Ritchie, M.E., et al., *limma powers differential expression analyses for RNA-sequencing and microarray studies.* Nucleic Acids Res, 2015. **43**(7): p. e47.

6. Chawade, A., E. Alexandersson, and F. Levander, *Normalyzer: a tool for rapid evaluation of normalization methods for omics data sets.* J Proteome Res, 2014. **13**(6): p. 3114-20.

7. Huber, W., et al., *Variance stabilization applied to microarray data calibration and to the quantification of differential expression.* Bioinformatics, 2002. **18 Suppl 1**: p. S96-104.

8. Alhamdoosh, M., Ng, M., Wilson, N.J., Sheridan, J. M., Huynh, H., Wilson, M.J., Ritchie, M. E. 2016. Combining multiple tools outperforms individual methods in gene set enrichment analyses. bioRxiv

Exp 8 FASP Methods – Orbitrap Exploris DIA

Protein samples were reduced, alkylated, and digested on-bead using filter-aided sample preparation [*Nature Methods* **6:** 359-62 (2009)] with sequencing grade modified porcine trypsin (Promega).

Tryptic peptides were trapped and eluted on 3.5um CSH C18 resin (Waters) (4mm x 75um) then separated by reverse phase XSelect CSH C18 2.5 um resin (Waters) on an in-line 150 x 0.075 mm column using an UltiMate 3000 RSLCnano system (Thermo). Peptides were eluted at a flow rate of 0.300uL/min using a 60 min gradient from 98% Buffer A:2% Buffer B to 95:5 at 2.0 minutes to 80:20 at 39.0 minutes to 60:40 at 48.0 minutes to 10:90 at 49.0 minutes and hold until 53.0 minutes and then equilibrated back to 98:2 at 53.1 minutes until 60 minutes. Eluted peptides were ionized by electrospray (2.4 kV) through a heated capillary (275°C) followed by data collection on an Orbitrap Exploris 480 mass spectrometer (Thermo Scientific).

Precursor spectra were acquired with a scan from 385-1015 Th at a resolution set to 60,000 with 100% AGC, max time of 50 msec, and an RF parameter at 40%. DIA was configured on the Orbitrap 480 to acquire 50 x 12 Th isolation windows at 15,000 resolution, normalized AGC target 500%, maximum injection time 40 ms). A second DIA was acquired in a staggered window (12 Th) pattern with optimized window placements.

Buffer A = 0.1% formic acid, 0.5% acetonitrile
Buffer B = 0.1% formic acid, 99.9% acetonitrile

Following data acquisition, data were searched using Spectronaut (Biognosys version 19.5) against the UniProt *Caenorhabditis elegans* database (June 2024) using the directDIA method with an identification precursor and protein q-value cutoff of 1%, generate decoys set to true, the protein inference workflow set to maxLFQ, inference algorithm set to IDPicker, quantity level set to MS2, cross-run normalization set to false, and the protein grouping quantification set to median peptide and precursor quantity. Protein MS2 intensity values were assessed for quality using ProteiNorm (Graw *et al.*). The data was normalized using VSN (Huber et al) and analyzed using proteoDA to perform statistical analysis using Linear Models for Microarray Data (limma) with empirical Bayes (eBayes) smoothing to the standard errors (Thurman et al, Ritchie et al). Proteins with an FDR adjusted p-value < 0.05 and a fold change > 2 were considered significant.

Acknowledgement:
IDeA National Resource for Quantitative Proteomics and NIH/NIGMS grant R24GM137786.

References:

1. Searle, B.C., Pino, L.K., Egertson, J.D. *et al.* Chromatogram libraries improve peptide detection and quantification by data independent acquisition mass spectrometry. *Nat Commun* **9,** 5128 (2018). https://doi.org/10.1038/s41467-018-07454-w

2. Graw S, Tang J, Zafar MK, Byrd AK, Bolden C, Peterson EC, and Byrum SD. proteiNorm – A user-friendly tool for normalization and analysis of TMT and label-free protein quantification. ACS Omega 2020 5(40), 25625-25633. DOI: 10.1021/acsomega.0c02564.

3. Thurman TJ, Washam CL, Alkam D, Bird JT, Gies A, Dhusia K, Robeson II MS, and Byrum SD. proteoDA: a package for quantitative proteomics. 2023. Journal of Open Source Software. DOI: 10.21105/joss.05184.

4. Bolstad, B., *preprocessCore: A collection of pre-processing functions.* 2019.

5. Ritchie, M.E., et al., *limma powers differential expression analyses for RNA-sequencing and microarray studies.* Nucleic Acids Res, 2015. **43**(7): p. e47.

6. Chawade, A., E. Alexandersson, and F. Levander, *Normalyzer: a tool for rapid evaluation of normalization methods for omics data sets.* J Proteome Res, 2014. **13**(6): p. 3114-20.

7. Huber, W., et al., *Variance stabilization applied to microarray data calibration and to the quantification of differential expression.* Bioinformatics, 2002. **18 Suppl 1**: p. S96-104.

8. Alhamdoosh, M., Ng, M., Wilson, N.J., Sheridan, J. M., Huynh, H., Wilson, M.J., Ritchie, M. E. 2016. Combining multiple tools outperforms individual methods in gene set enrichment analyses. bioRxiv
