# Supplementary material for: Integrating endogenous TurboID and data-independent acquisition mass spectrometry for in vivo proximity labeling
Source: EMBO J. 2025 Dec 11;45(2):592–632. doi: 10.1038/s44318-025-00660-5 (PMC12811337; doi:10.1038/s44318-025-00660-5)
Supplement: Supplementary file 17 — Source data Fig. 1 [file 44318_2025_660_MOESM17_ESM.zip › Figure 1/1D-E/README.rtf]

This file contains western blot data for Figure 1D and 1E.
