# Supplementary material for: Integrating endogenous TurboID and data-independent acquisition mass spectrometry for in vivo proximity labeling
Source: EMBO J. 2025 Dec 11;45(2):592–632. doi: 10.1038/s44318-025-00660-5 (PMC12811337; doi:10.1038/s44318-025-00660-5)
Supplement: Supplementary file 18 — Source data Fig. 2 [file 44318_2025_660_MOESM18_ESM.zip › Figure 2/2B/README.rtf]

Source Data for Figure 2B are in multiple files.Original data for Experiments 1 through 8 are in SourceDataForFigure2B_1.xls through SourceDataForFigure2B_8.xls, respectively. SourceDataForFigure2B_9.xls contains additional calculationsThese 9 files are also included as DatasetsEV1.xls through DatasetsEV9.xls.
