# Supplementary material for: Integrating endogenous TurboID and data-independent acquisition mass spectrometry for in vivo proximity labeling
Source: EMBO J. 2025 Dec 11;45(2):592–632. doi: 10.1038/s44318-025-00660-5 (PMC12811337; doi:10.1038/s44318-025-00660-5)
Supplement: Supplementary file 19 — Source data Fig. 3 [file 44318_2025_660_MOESM19_ESM.zip › Figure 3/3A-E/README.rtf]

Source Data for Figure 3 are in multiple files, with the corresponding Experiment number shown as the last digit of the file nameOriginal data for Experiment 1 (panels A and B) is in SourceDataForFigure3A-E_1.xlsOriginal data for Experiment 3 (panel E) is in SourceDataForFigure3A-E_3.xlsOriginal data for Experiment 6 (panels A and C) is in SourceDataForFigure3A-E_6.xlsOriginal data for Experiment 8 (panel D) is in SourceDataForFigure3A-E_8.xlsThese 4 files are also included as DatasetsEV1.xls, DatasetsEV3.xls, DatasetsEV6.xls, and DatasetsEV8.xls, respectively.
