# Supplementary material for: Integrating endogenous TurboID and data-independent acquisition mass spectrometry for in vivo proximity labeling
Source: EMBO J. 2025 Dec 11;45(2):592–632. doi: 10.1038/s44318-025-00660-5 (PMC12811337; doi:10.1038/s44318-025-00660-5)
Supplement: Supplementary file 21 — Source data Fig. 5 [file 44318_2025_660_MOESM21_ESM.zip › Figure 5/5A-D/README.rtf]

Source Data for Figure 5 are in multiple files.The Venn diagram and scatterplot data are in SourceDataForFigure5A-D.xls. This file is also included as DatasetsEV10.xls.The original western blots for Figure 5C are in SourceDataForFigure5C_WB.pdf (see replicate 2).The original western blots for Figure 5D are in SourceDataForFigure5D_WB.pdf (see replicate 2).
