# Supplementary material for: Integrating endogenous TurboID and data-independent acquisition mass spectrometry for in vivo proximity labeling
Source: EMBO J. 2025 Dec 11;45(2):592–632. doi: 10.1038/s44318-025-00660-5 (PMC12811337; doi:10.1038/s44318-025-00660-5)
Supplement: Supplementary file 21 — Source data Fig. 5 [file 44318_2025_660_MOESM21_ESM.zip › Figure 5/5A-D/EMBOJ-2025-122132_SourceDataForFigure5D_WB.pdf]

Experiment 7

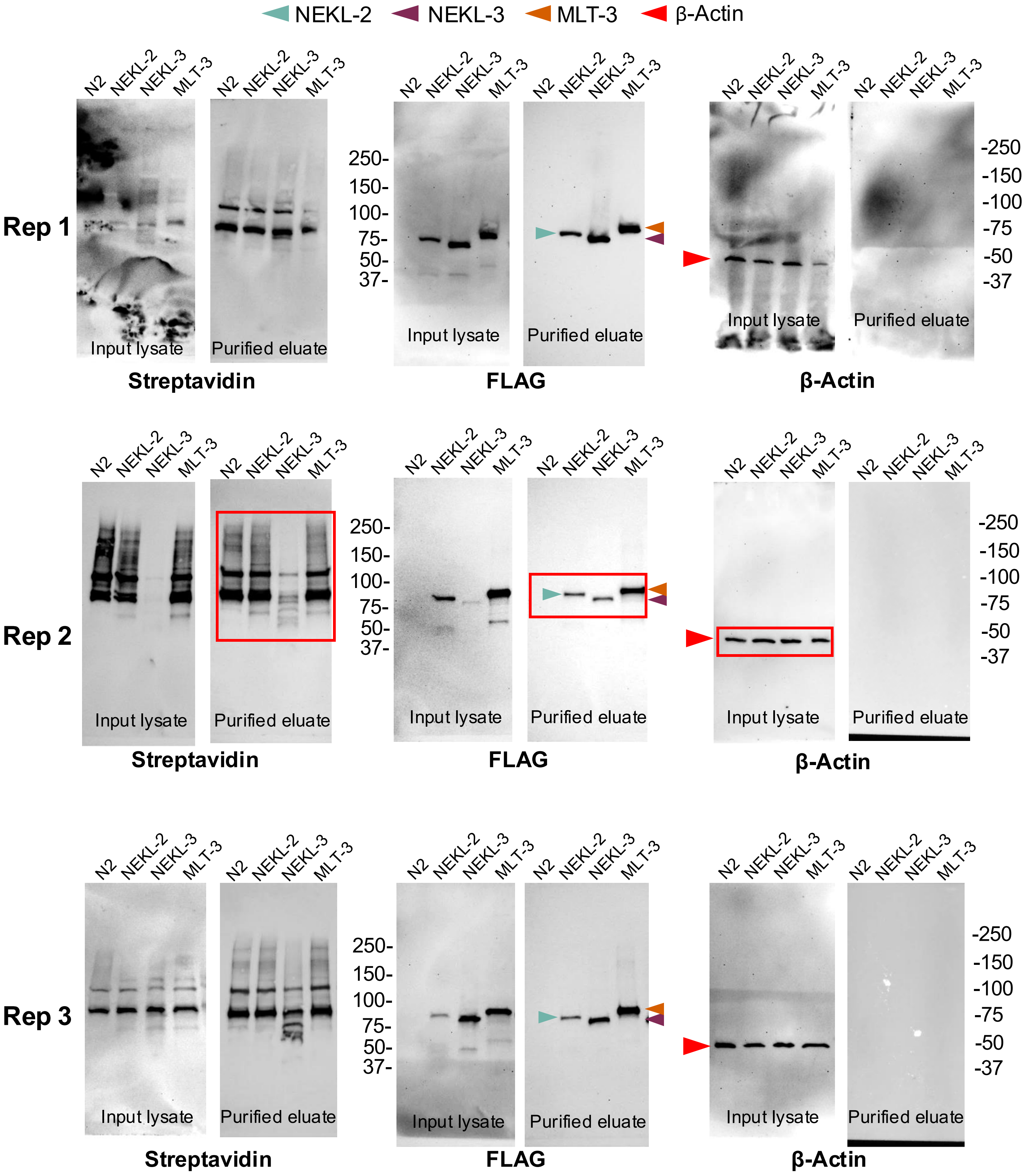

PYC-1a, 129.3 kDa; PYC-1b, 67.8 kDa; PCCA-1, 79.7 kDa; MCCC-1, 73.7 kDa; POD-2a, 230.6 kDa; POD-2b, 91.4 kD

CT-TurboID: NEKL-2 80.9 kDa; NEKL-3 75.0 kDa; MLT-3 83.5 kDa
