# Supplementary material for: Integrating endogenous TurboID and data-independent acquisition mass spectrometry for in vivo proximity labeling
Source: EMBO J. 2025 Dec 11;45(2):592–632. doi: 10.1038/s44318-025-00660-5 (PMC12811337; doi:10.1038/s44318-025-00660-5)
Supplement: Supplementary file 26 — Expanded View Figures [file 44318_2025_660_MOESM26_ESM.pdf]

Expanded View Figures

Figure EV1. AlphaFold modeling of NEKL–MLT interactions.

(**A–E**) AlphaFold3 modeling and ChimeraX rendering of predicted (**A**) NEKL-3–MLT-3, (**B**) MLT-2–MLT-4–NEKL-2, (**C**) MLT-2–MLT-3, (**D**) NEKL-2–NEKL-3, and (**E**) MLT-3–NEKL-3–NEKL-2–MLT-2–MLT-4 protein complexes. Black lines between proteins indicate high-confidence Predicted Aligned Error (PAE) pseudobonds (PAE ≤ 6 Å; interatomic-distance ≤5 Å). For clarity, highly unstructured portions of the peptides (B-factor ≤30) were hidden. (**A–E**) Show protein-backbone ribbon diagrams (colored by peptide) along with single-color (green) PAE diagrams indicating regions of high structural confidence (low PAE) within and between the indicated proteins. Dashed-line cyan boxes correspond to regions where the two proteins are predicted for form a coherent structural unit. (**A–D**) Additionally show protein ribbon diagrams colored by PAE domain along with corresponding color-matched PAE diagrams. Ribbon diagrams in (**C–E**) also show partially transparent surface renderings. Inter-chain predicted Template Modeling (ipTM) values, and Local Interaction Scores (LIS) are shown for the indicated proteins modeled as heterodimers by AlphaFold3. Note that the five-protein complex (**E**) predicts very similar protein–protein interfaces as the subcomplex models, consistent with the hypothesis that the two subcomplexes can physically interact.

© The Author(s)

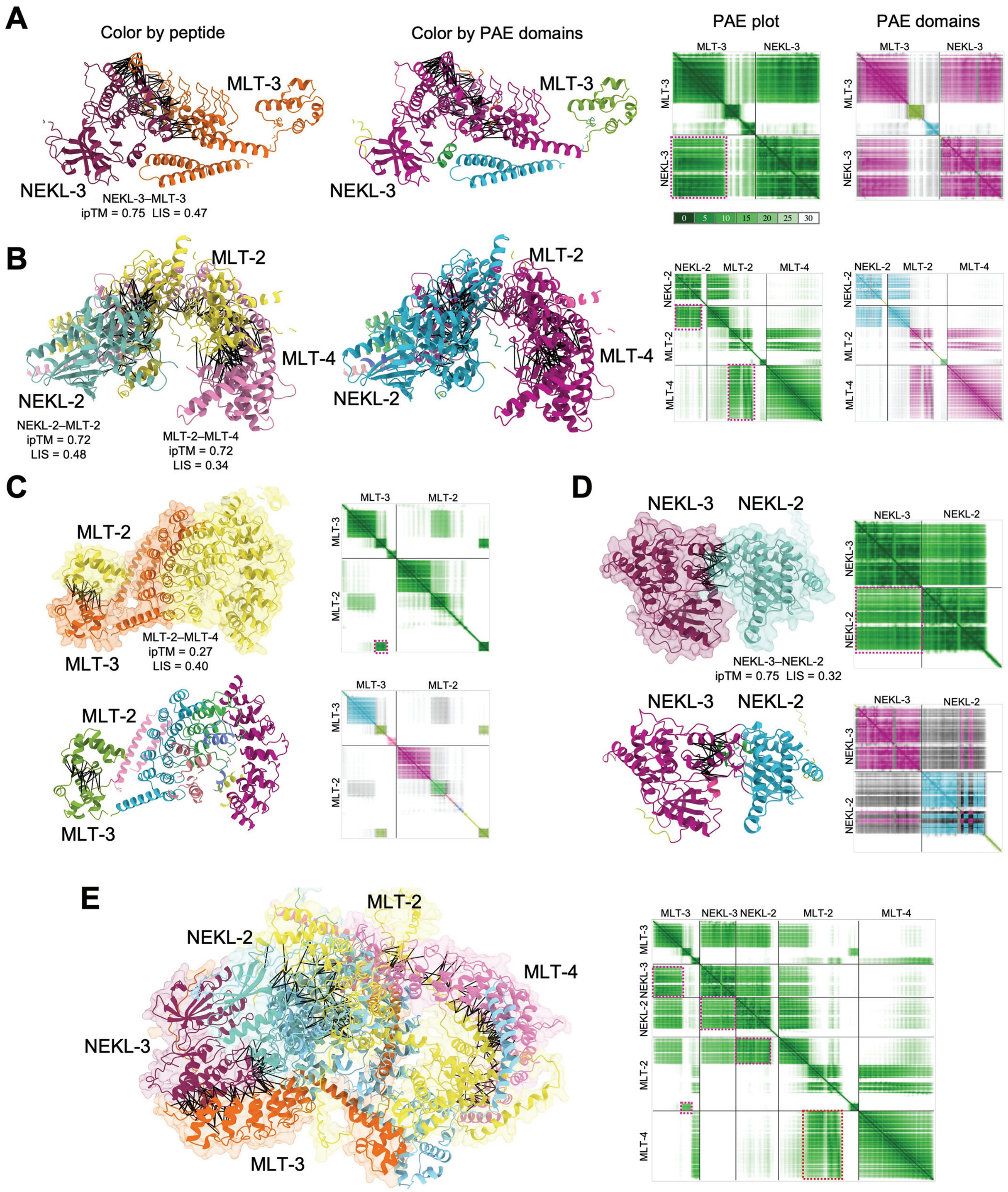
A
Color by peptide
Color by PAE domains
PAE plot
PAE domains
MLT-3
NEKL-3
NEKL-3–MLT-3
ipTM = 0.75 LIS = 0.47
0 5 10 15 20 25 30
B
MLT-2
MLT-4
NEKL-2
NEKL-2–MLT-2
ipTM = 0.72
LIS = 0.48
MLT-2–MLT-4
ipTM = 0.72
LIS = 0.34
C
MLT-2
MLT-3
MLT-2–MLT-4
ipTM = 0.27
LIS = 0.40
D
NEKL-3
NEKL-2
NEKL-3–NEKL-2
ipTM = 0.75 LIS = 0.32
E
MLT-2
NEKL-2
MLT-4
NEKL-3
MLT-3

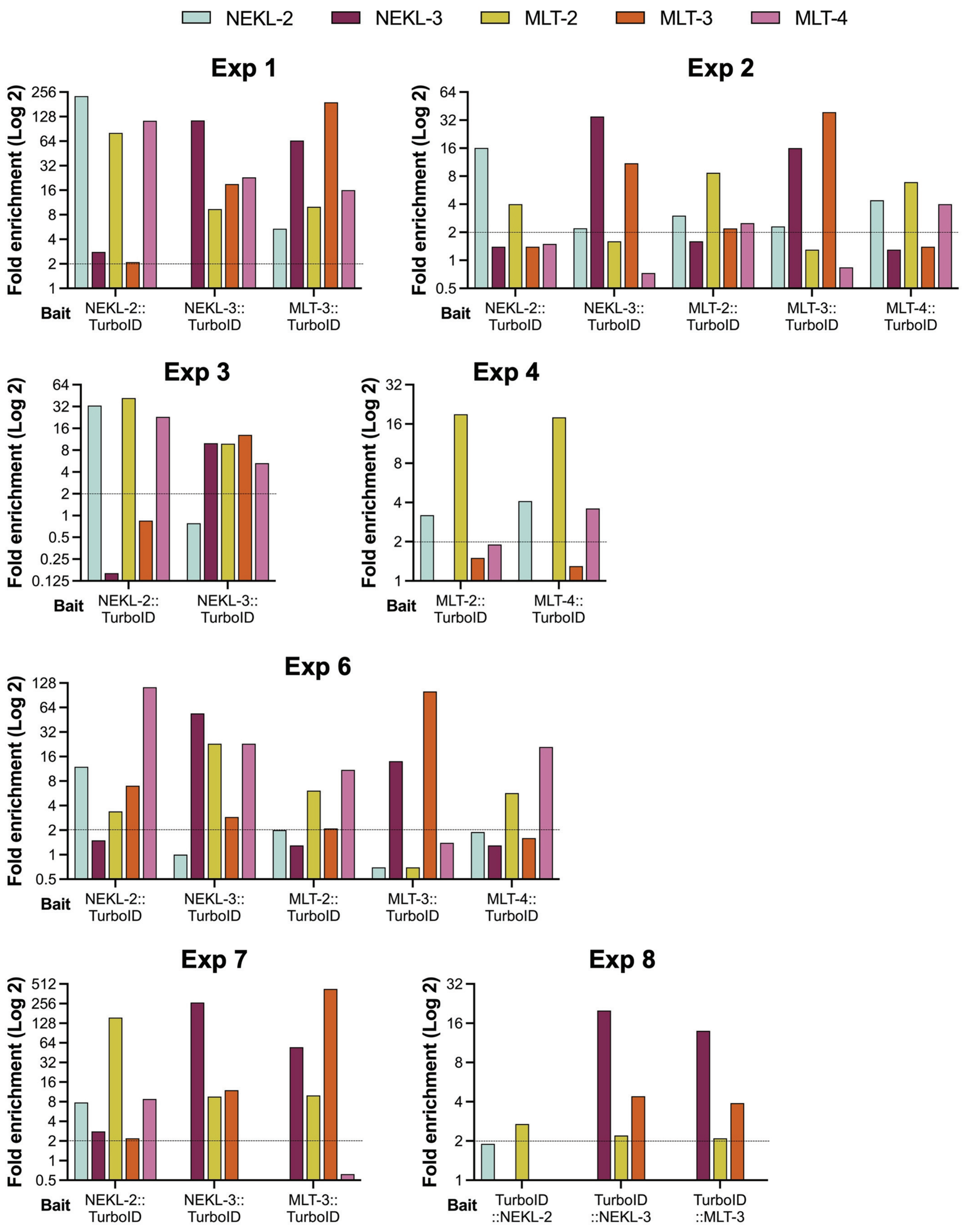
NEKL-2
NEKL-3
MLT-2
MLT-3
MLT-4
Exp 1
Exp 2
Exp 3
Exp 4
Exp 6
Exp 7
Exp 8
Fold enrichment (Log 2)
Bait
NEKL-2:: TurboID
NEKL-3:: TurboID
MLT-2:: TurboID
MLT-3:: TurboID
MLT-4:: TurboID
TurboID ::NEKL-2
TurboID ::NEKL-3
TurboID ::MLT-3
512
256
128
64
32
16
8
4
2
1
0.5
0.25
0.125

 © The Author(s)

Figure EV2. Fold changes of NEKL-MLT bait and prey proteins.

Bar graphs of fold changes shown in Fig. 2B and Datasets EV1–8 for Exp 1–4 and 6–8.

© The Author(s)

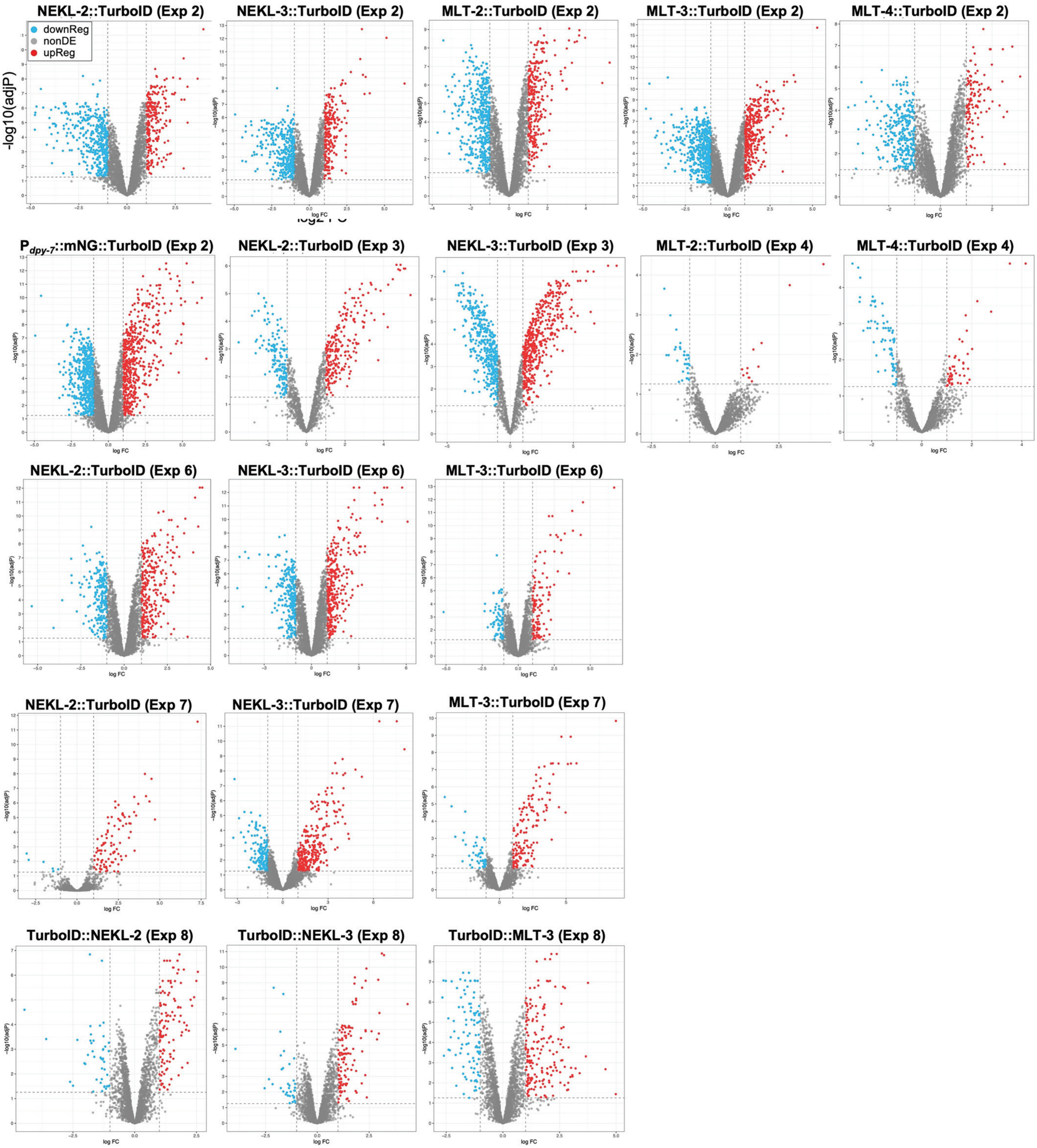

Figure EV3. Volcano plots.

Volcano plots of bait–TurboID strains versus N2 control showing the $\log_2$ fold change (FC) versus $-\log_{10}$ adjusted *P* value. Red and blue dots indicate proteins that were up and down, respectively, in the bait strain relative to the N2 strain; select identified bait and prey proteins are indicated by arrows. NonDE, not differentially expressed. Red and blue dots indicate proteins that were up in the bait and N2 strains, respectively. Additional volcano plots are shown in Figs. 3A, 6B, EV5, and EV7. Data corresponding to volcano plots are available in Datasets EV1–8.

 © The Author(s)

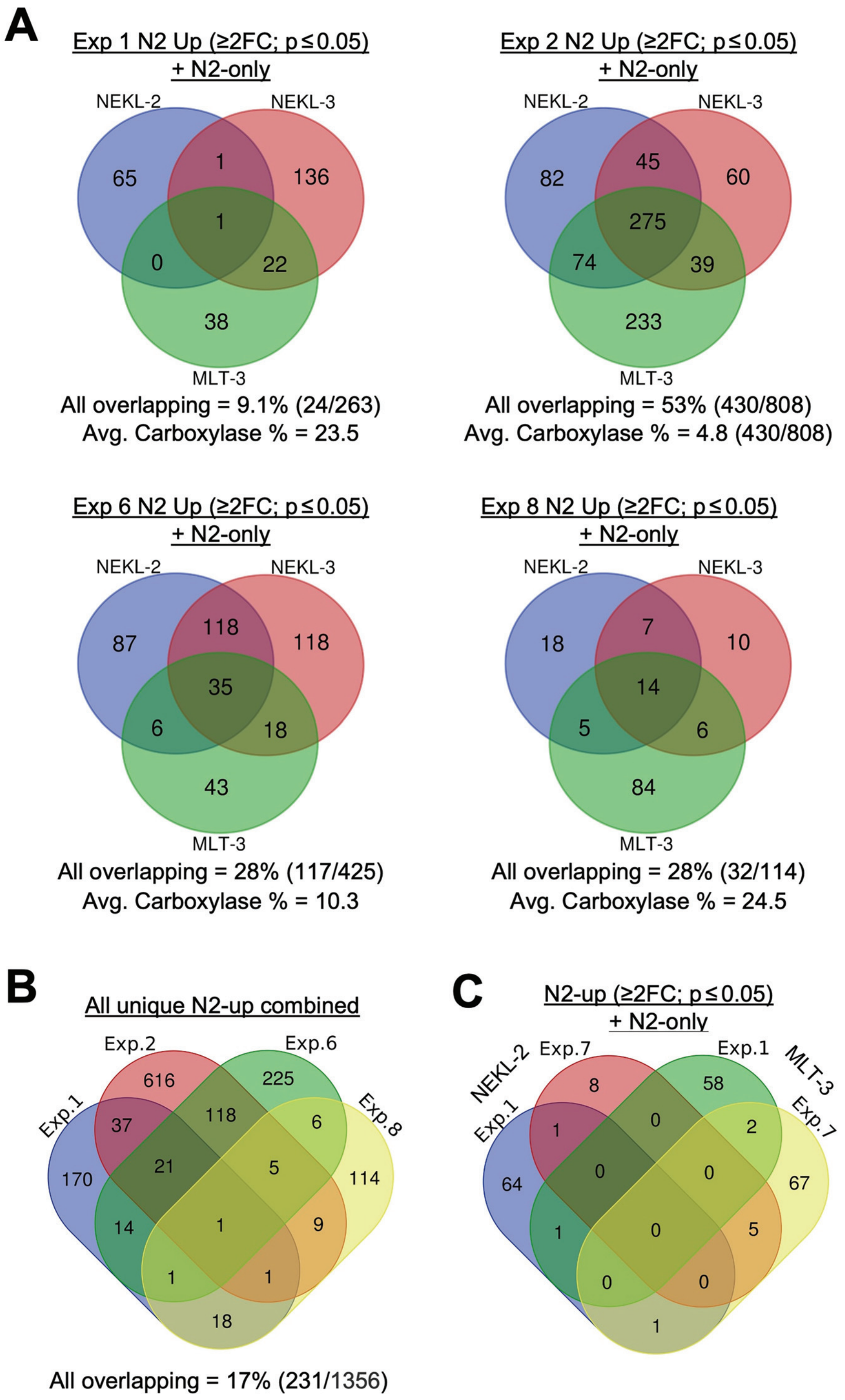

Figure EV4. Analysis of N2-enriched proteins.

(**A**) Venn diagrams showing overlap of proteins enriched (≥2-fold (FC); adjusted $P \leq 0.05$) in N2 samples (N2 Up) versus NEKL-2::TurboID, NEKL-3::TurboID, and MLT-3::TurboID bait samples from Exp 1, Exp 2, Exp 6, and Exp 8. N2-only indicates proteins that were present in all technical replicates of the N2 sample but were absent in all technical replicates of the corresponding bait–TurboID; Also see Fig. 2B. Note variability in the extent of overlap among experiments. (**B**) Venn diagram comparing overlaps among the combined N2 Up proteins from Exp 1, Exp 2, Exp 6, and Exp 8. Note the relative lack of consistently overlapping proteins among experiments. (**C**) Venn diagram showing overlaps of N2-enriched proteins from NEKL-2::TurboID and MLT-3::TurboID (Exp 1 and Exp 7). Little overlap was observed between comparable biological replicates of NEKL-2::TurboID and MLT-3::TurboID as well as between the two bait proteins within the same biological replicate.

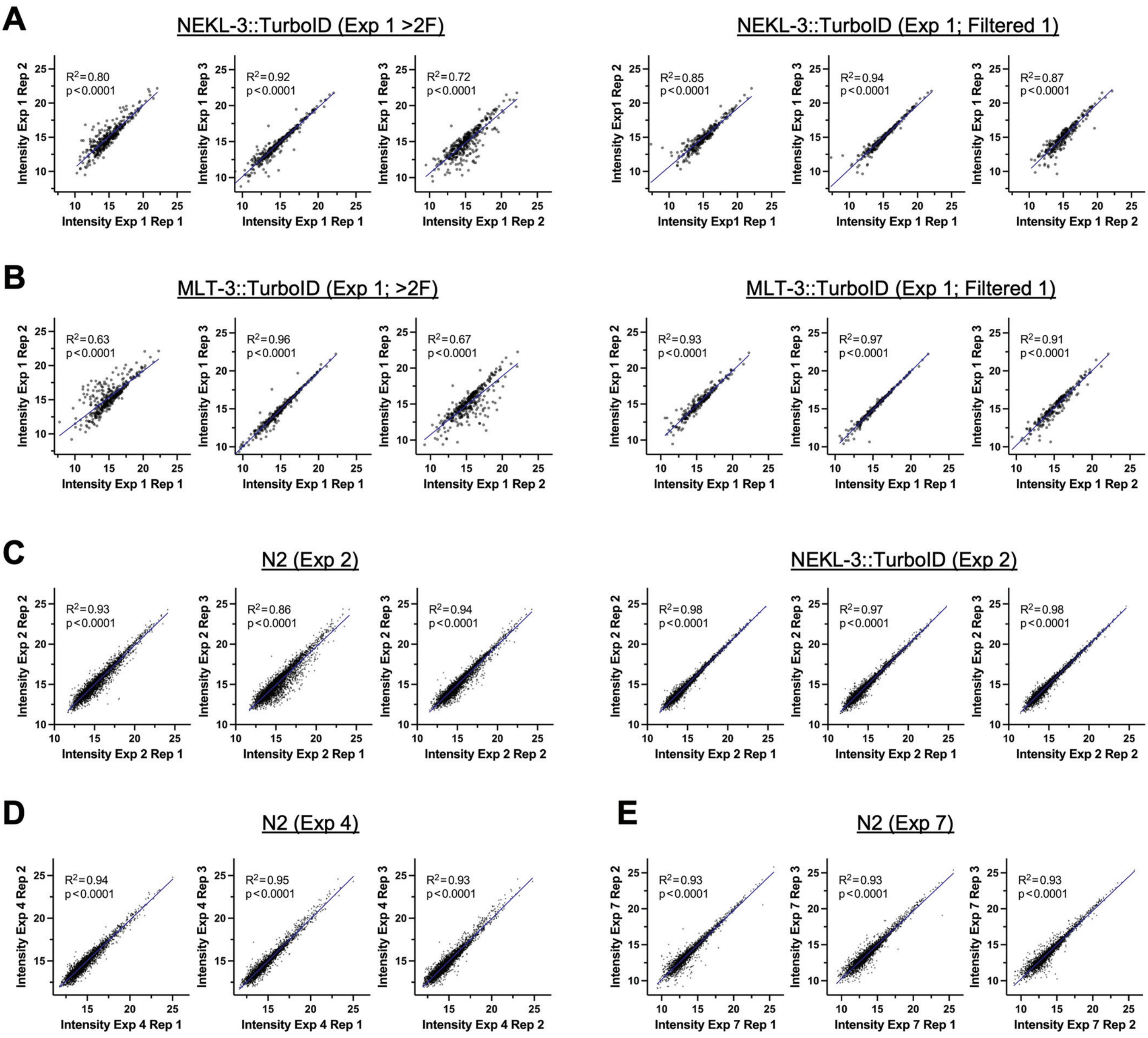

Figure EV5. Additional analysis of technical replicates.

(A–E) Scatter plot diagrams showing correlations of individual protein intensities (abundance) between the indicated replicate pairs. R^2 values were derived using simple linear regression; raw data are available in Dataset EV10. Also see Fig. 5.

 © The Author(s)

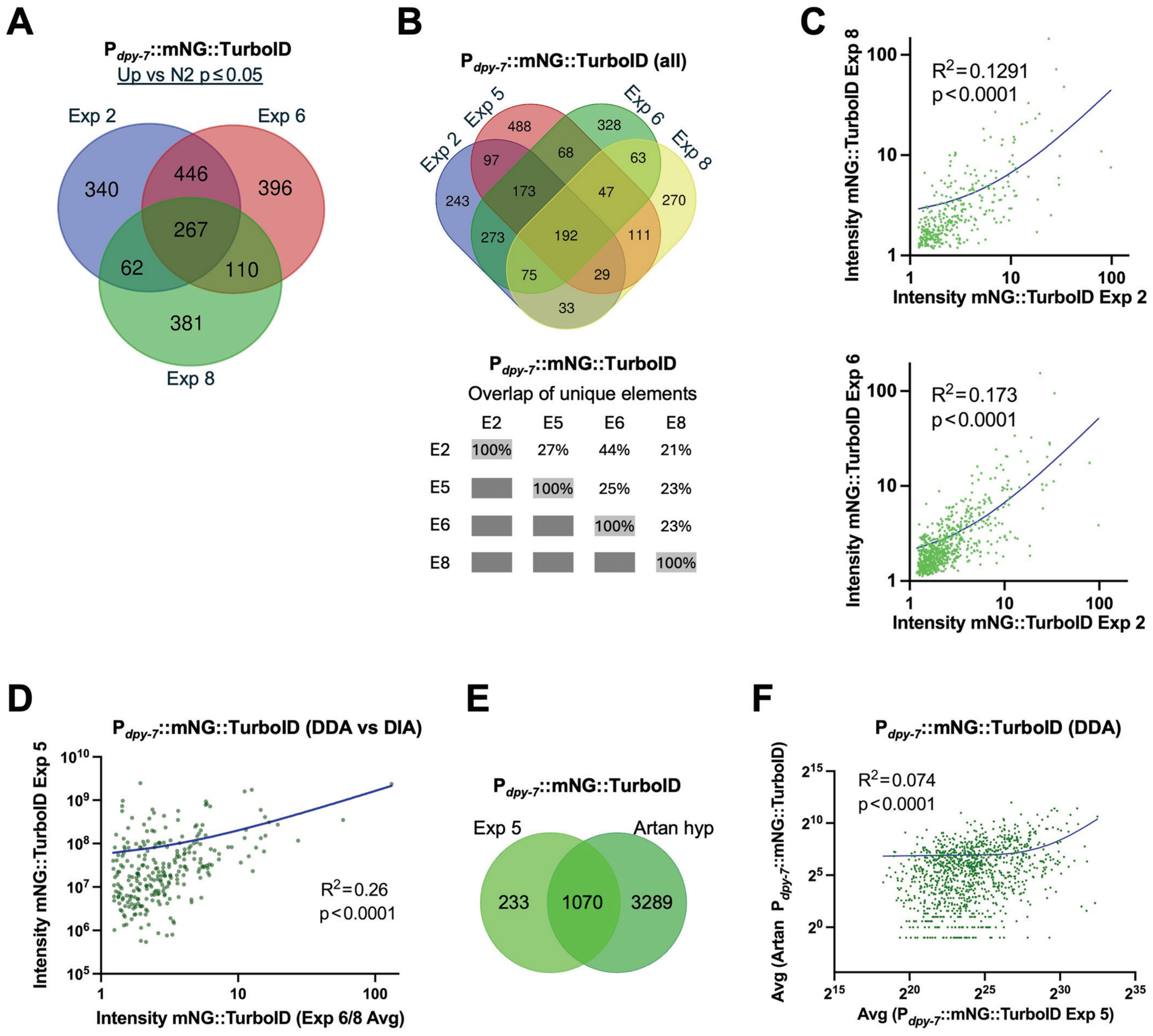

Figure EV6. Additional analysis of P_{dpy-7}::mNG::TurboID datasets.

(**A**) Venn diagram overlap of proteins significantly enriched (based on adjusted *P* values) in the three DIA-MS P_{dpy-7}::mNG::TurboID experiments. (**B**) Venn diagram and quantification of pairwise overlaps for the four P_{dpy-7}::mNG::TurboID experiments. Proteins included were detected in all technical replicates. Note that although Exp 2 and Exp 6 showed the greatest overlap in detected proteins, the correlation in protein levels was strongest between Exp 6 and Exp 8 (also see Fig. 6C). (**C**) Scatter plots showing correlations of protein intensities (abundance) for the mNG::TurboID control strain from indicated experimental pairs. R^2 values were derived using simple linear regression; raw data are available in Dataset EV11. The curved regression lines are due to the conversion of axes to a log scale. The markedly lower R^2 values, relative to Exp 6 vs Exp 8, are due in large part to discrepancies in intensity values at the high end of the scale. (**D**) Scatter plot showing weak correlation between Exp 5 (DDA-MS) and averaged data from Exp 6 and Exp 8 (DIA-MS). (**E**) Overlap and (**F**) correlation between Exp 5 and DDA-MS experiment from Artan and colleagues (PMID: 35665632). Note that although the proteins detected in Exp 5 overlapped extensively with a subset of those previously reported, the correlation in their expression levels was weak.

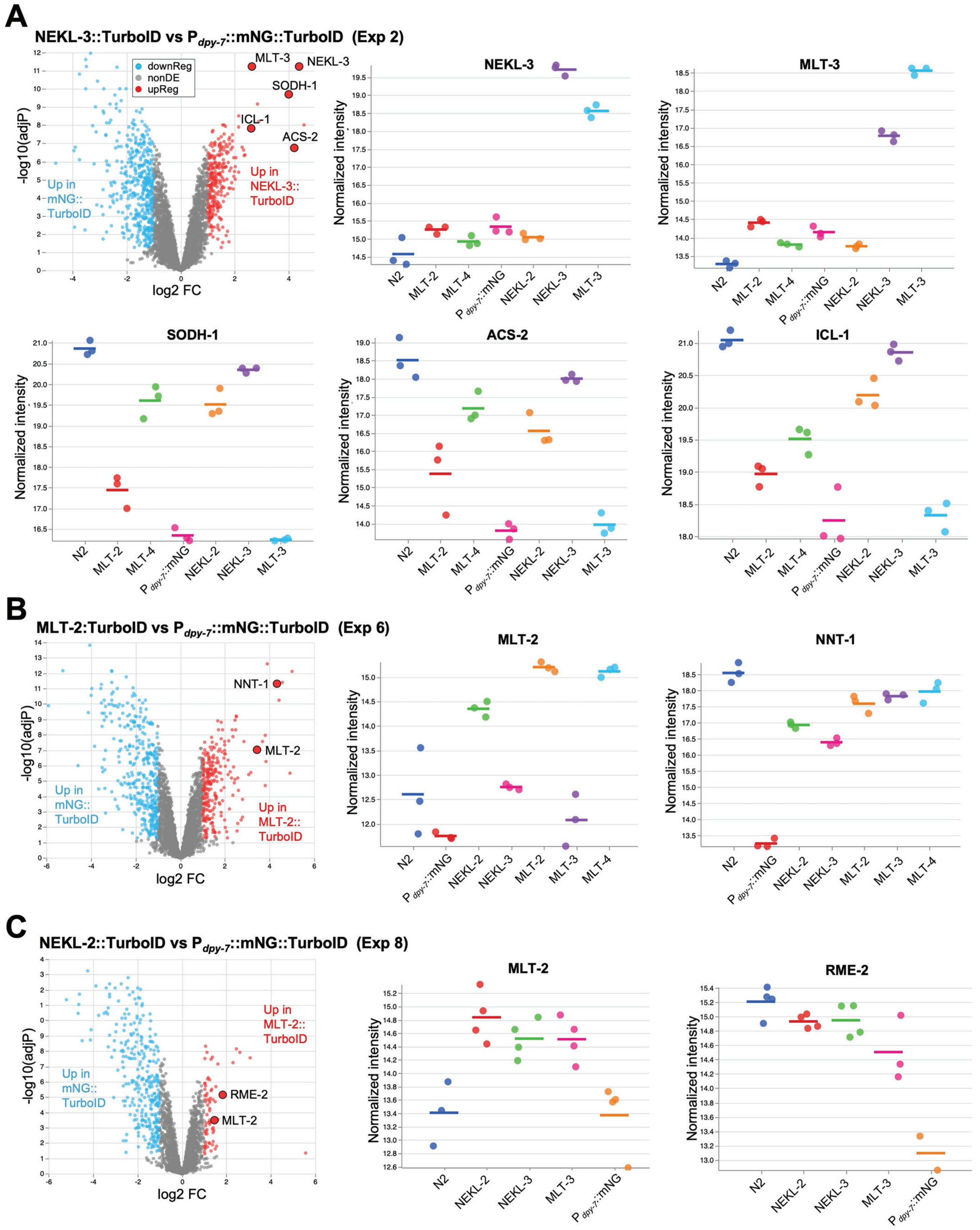
A
NEKL-3::TurboID vs P dpy-7::mNG::TurboID (Exp 2)
downReg
nonDE
upReg
MLT-3
NEKL-3
SODH-1
ICL-1
ACS-2
Up in mNG:: TurboID
Up in NEKL-3:: TurboID
-log10(adjP)
log2 FC
NEKL-3
MLT-3
SODH-1
ACS-2
ICL-1
Normalized intensity
N2
MLT-2
MLT-4
P dpy-7::mNG
NEKL-2
NEKL-3
MLT-3
B
MLT-2:TurboID vs P dpy-7::mNG::TurboID (Exp 6)
NNT-1
MLT-2
Up in mNG:: TurboID
Up in MLT-2:: TurboID
-log10(adjP)
log2 FC
MLT-2
NNT-1
Normalized intensity
N2
P dpy-7::mNG
NEKL-2
NEKL-3
MLT-2
MLT-3
MLT-4
C
NEKL-2::TurboID vs P dpy-7::mNG::TurboID (Exp 8)
RME-2
MLT-2
Up in mNG:: TurboID
Up in MLT-2:: TurboID
-log10(adjP)
log2 FC
MLT-2
RME-2
Normalized intensity
N2
NEKL-2
NEKL-3
MLT-3
P dpy-7::mNG

 © The Author(s)

Figure EV7. P$_{dpy-7}$::mNG::TurboID down proteins.

(**A–C**) Volcano plots of P$_{dpy-7}$::mNG::TurboID versus NEKL-3::TurboID (**A**), MLT-2::TurboID (**B**), and NEKL-2 TurboID (**C**) indicating the $\log_2$ fold change (FC) versus $-\log_{10}$ adjusted *P* values. Enlarged red dots indicate proteins shown in the accompanying intensity plots. Intensity plots ($\log_2$ scale) showing the abundance of the indicated bait and prey proteins; individual dots represent values from the three technical replicates. For SODH-1, ACS-2, ICL-1, NNT-1, and RME-2, levels in P$_{dpy-7}$::mNG::TurboID were markedly lower than in the N2 or most bait–TurboID strains.

© The Author(s)

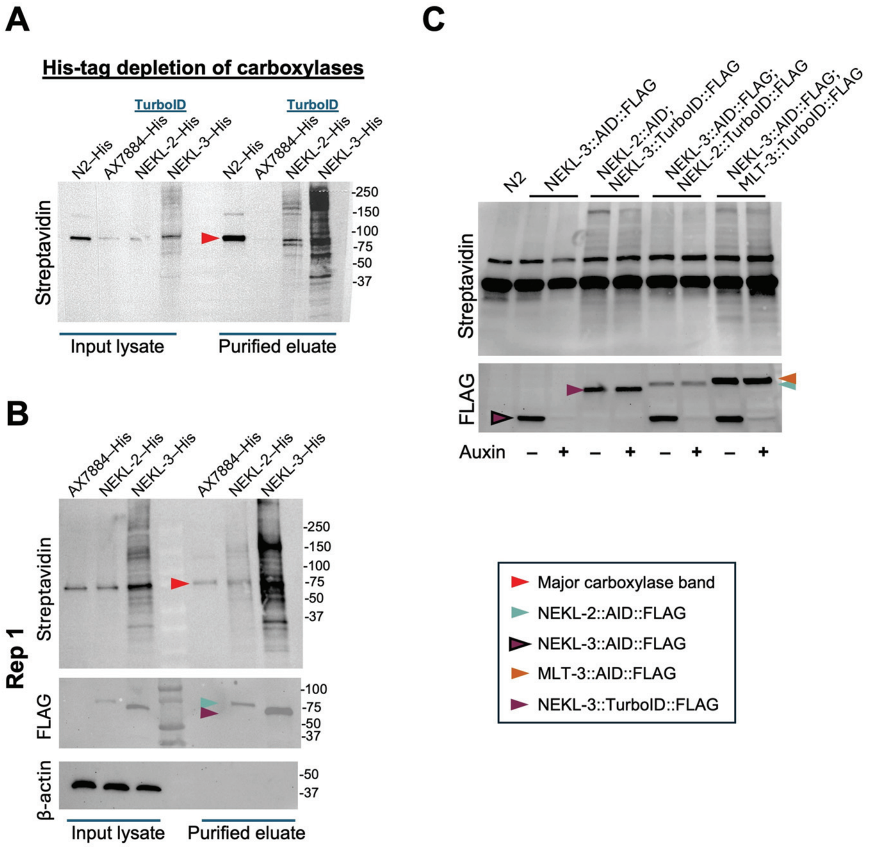

Figure EV8. Methodological variations.

(**A–C**) Western blots with specific proteins indicated by the key. (**A**) Streptavidin-probed western blot showing a reduction in the major carboxylase band in strains that underwent carboxylase depletion (AX7884, NEKL-2, and NEKL-3). –His, lysates were incubated with nickel-coated (His-binding) beads to remove the endogenous His-tagged versions of PYC-1, PCCA-1, MCCC-1, and POD-2. N2 does not encode His-tagged carboxylases, accounting for the stronger band in this strain. Increased protein concentration in the eluate lanes facilitates the visualization of the bait-induced biotinylated bands. (**B**) Western blots from Exp 3, replicate 1 Exp showing biotinylated products (streptavidin), bait proteins (FLAG), and loading control (β-actin). (**C**) Western blot of lysates from the indicated strains after incubation in the presence or absence of auxin, which leads to the degradation of the AID-tagged proteins. Note that the NEKL-2::AID strain contained a defective FLAG tag and was thus not detectable.

 © The Author(s)

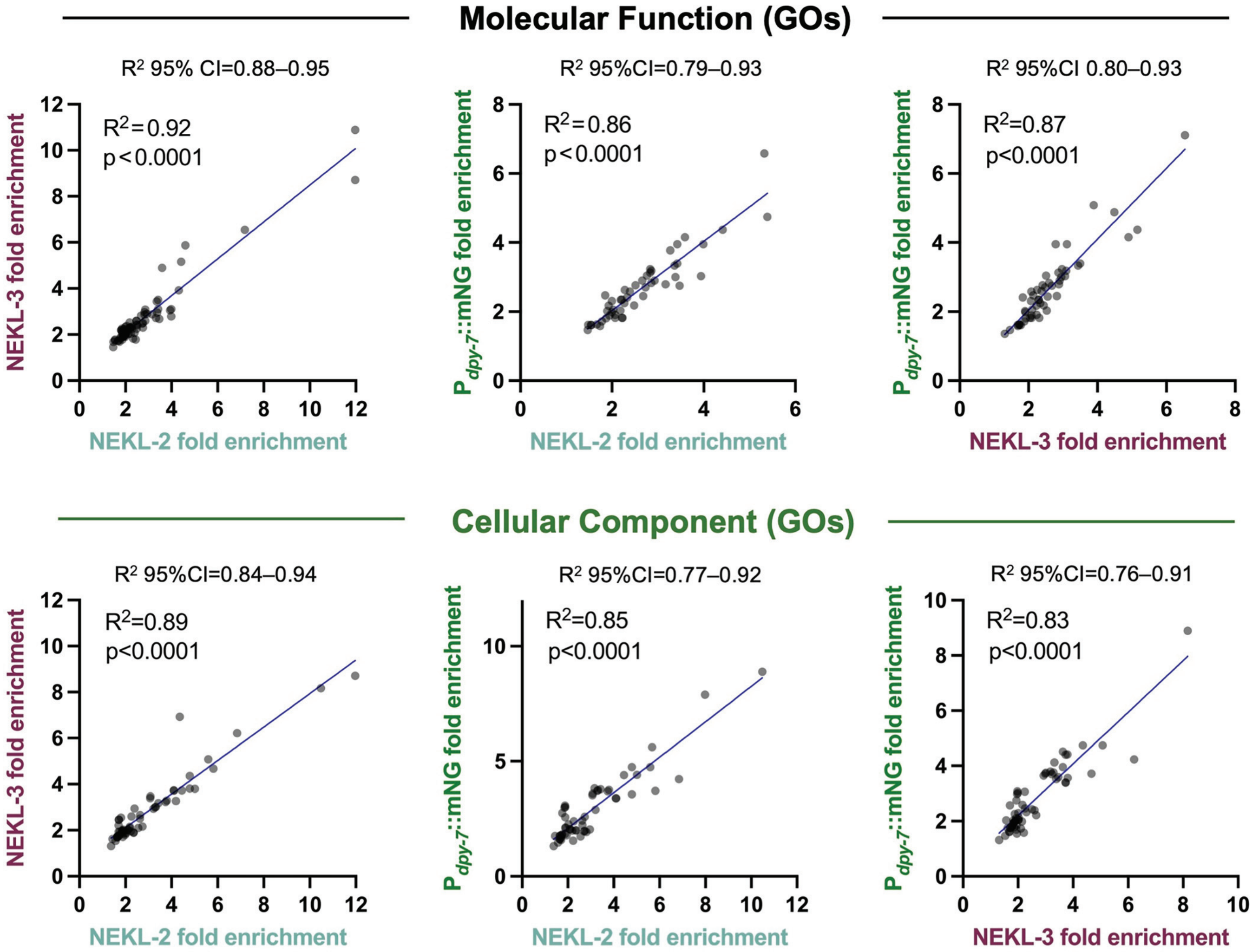

Figure EV9. Correlations of GO terms.

Scatterplots showing correlations in fold enrichments of Molecular Function and Cellular Component GOs for the combined NEKL-2::TurboID, NEKL-3::TurboID, and $P_{dpy\text{-}7}$::mNG::TurboID datasets. 95%CI, 95% confidence interval. Although correlation was greatest between NEKL-2–NEKL-3, this was not statistically significant based on CIs.
